# Co-Amorphous Screening for the Solubility Enhancement of Poorly Water-Soluble Mirabegron and Investigation of Their Intermolecular Interactions and Dissolution Behaviors

**DOI:** 10.3390/pharmaceutics10030149

**Published:** 2018-09-05

**Authors:** Ji-Hun An, Changjin Lim, Alice Nguvoko Kiyonga, In Hwa Chung, In Kyu Lee, Kilwoong Mo, Minho Park, Wonno Youn, Won Rak Choi, Young-Ger Suh, Kiwon Jung

**Affiliations:** 1 Institute of Pharmaceutical Sciences, College of Pharmacy, CHA University, Sungnam 13844, Korea; ajh@chauniv.ac.kr (J.-H.A.); koryoi0709@gmail.com (C.L.); gabriella@chauniv.ac.kr (A.N.K.); minho.park92@gmail.com (M.P.); ygsuh@cha.ac.kr (Y.-G.S.); 2 R&D Center, Sungwun Pharmacopia, Seoul 05836, Korea; ihchung7@sungwun.net (I.H.C.); platon777@sungwun.net (I.K.L.); chemrnd@sungwun.net (K.M.); 3 College of Pharmacy and Medical Research Center, Chungbuk National University, Chungbuk 28644, Korea; wonno80@naver.com (W.Y.); cwonr@naver.com (W.R.C.)

**Keywords:** mirabegron, co-amorphous, active pharmaceutical ingredient, aqueous solubility, ionic interaction

## Abstract

In the present study, the screening of Mirabegron (MBR) co-amorphous was performed to produce water-soluble and thermodynamically stable MBR co-amorphous with the purpose of overcoming the water solubility problem of MBR. MBR is Biopharmaceutics Classification System (BCS) class II drug used for the treatment of an overreactive bladder. The co-amorphous screening was carried out by means of the vacuum evaporation crystallization technique in methanol solvent using three water-soluble carboxylic acids, characterized by a pKa difference greater than 3 with MBR such as fumaric acid (FA), l-pyroglutamic acid (PG), and citric acid (CA). Powder X-ray diffraction (PXRD) results suggested that all solid materials produced at MBR-FA (1 equivalent (eq.)/1 equivalent (eq.)), MBR-PG (1 eq./1 eq.), and MBR-CA (1 eq./1 eq.) conditions were amorphous state solid materials. Furthermore, by means of solution-state nuclear magnetic resonance (NMR) (^1^H, ^13^C, and 2D) and attenuated total reflection Fourier transform infrared (ATR-FTIR) spectroscopy, we could assess that MBR and carboxylic acid molecules were linked via ionic interactions to produce MBR co-amorphous. Besides, solid-state cross polarization (CP)/magic angle spinning (MAS) ^13^C-NMR analysis was conducted for additional assessment of MBR co-amorphous. Afterwards, dissolution tests of MBR co-amorphouses, MBR crystalline solid, and MBR amorphous were carried out for 12 h to evaluate and to compare their solubilities, dissolution rates, and phase transformation phenomenon. Here, the results suggested that MBR co-amorphouses displayed more than 57-fold higher aqueous solubility compared to MBR crystalline solid, and PXRD monitoring result suggested that MBR co-amorphouses were able to maintain their amorphous state for more than 12 h. The same results revealed that MBR amorphous exhibited increased solubility of approximatively 6.7-fold higher compared to MBR crystalline solid. However, the PXRD monitoring result suggested that MBR amorphous undergo rapid phase transformation to crystalline form in just 35 min and that within an hour all MBR amorphous are completely converted to crystalline solid. Accordingly, the increase in MBR co-amorphous’ solubility was attributed to the presence of ionic interactions in MBR co-amorphous molecules. Moreover, from the differential scanning calorimetry (DSC) monitoring results, we predicted that the high glass transition temperature (*T_g_*) of MBR co-amorphous compared to MBR amorphous was the main factor influencing the phase stability of MBR co-amorphous.

## 1. Introduction

Solid-state drug substances can be classified either as crystalline solid or as amorphous solid. Amorphous solid refers to solid materials characterized by short-range order (intermolecular interactions) but devoid of long-range order (molecular packing) particularity of crystalline solids. Amorphous solids possess higher thermodynamic energy than crystalline solids owing to the exhibition of great solubility and high dissolution rate [1,2]. Accordingly, various studies have suggested the preparation of crystalline drugs into amorphous materials with the purpose of improving the water solubility of the drugs. Nevertheless, due to their weak thermodynamic stability, amorphous solids rapidly convert to less soluble crystalline solids [1,2,3].

As one solution to increase the stability of amorphous solids to increase water solubility, polymers have been employed during amorphous drugs formulation to prevent their conversion to crystalline solids and, consequently, to maintain their amorphous state. This can be also called polymer-based amorphous solid-dispersions (ASD). The combination of ASD with polymer is assumed to elevate the glass transition temperature (*T_g_*) of the amorphous drug, which thereby prevents the drug‘s phase transformation and subsequently promotes the drug’s amorphous state stability [4,5,6,7]. However, barriers to improving the solubility of ASD drugs exist due to the characteristics of ASD enclosing which stabilizes the amorphous drug [8,9]. Besides, limitations including the decrease of amorphous solids’ *T_g_* values and consequently the promotion of their phase transformation to crystalline form have been associated with hygroscopic properties of the polymers [10]. Thus, investigation of new alternatives to polymers, capable of promoting the thermodynamic stabilities of amorphous state drugs, is strongly needed.

Co-amorphous designates two or more low weight molecules interacting to form homogenous amorphous single-phase system. Co-amorphous solids are assumed to improve the drug’s dissolution rate due to their ability to stabilize the drug’s amorphous state. These co-amorphous solids possess high *T_g_* values, which are presumed to promote their thermodynamic stability and to prevent their recrystallization process to crystalline solids during the drug formulation [11,12,13,14]. Also, co-amorphous solids have unique properties such as high aqueous solubility and dissolution rate, and improved thermodynamic stability because they are characterized by short-range order [14]. Co-amorphous solids can be formed via ionic interactions [15,16], hydrogen bonding [17,18,19], and π–π interactions [20,21] between two or more low molecular components.

**Figure 1:**
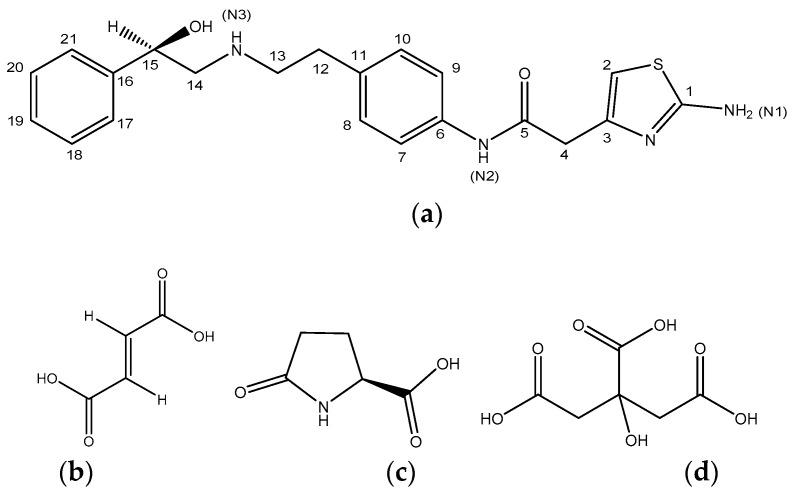
Chemical structures and carbon numbering for: (**a**) Mirabegron; (**b**) fumaric acid (FA); (**c**) l-Pyroglutamic acid (PG); (**d**) citric acid (CA).

Mirabegron (MBR, Figure 1a) is an active pharmaceutical ingredient (API) used for the treatment of an overactive bladder and belongs to beta-3 adrenergic receptor agonist class. Developed by Astellas pharma, MBR is marketed under the brand name Betmiga^®^ [22,23,24]. MBR exists under its crystalline form and has been classified as BCS class II compound. Because of its poor aqueous solubility, 0.082 mg/mL, MBR displays a low bioavailability of approximatively 29% [25]. Hence, to improve MBR solubility and bioavailability, water-soluble polymer, namely polyethylene glycol (PEG) 8000, has been employed during MBR drugs formulation [26]. However, PEG has been reported to damage the mucous membranes. Thus, with the purpose of restraining the use of PGE 8000, several studies have focused on improving the aqueous solubility of MBR though the preparation of MBR’s salt form [27,28], solvate [28,29], and ASD [25]. However, solubility and dissolution data related to MRB solids are assumed insignificant.

In the present study, the screening of MBR co-amorphous was carried out using vacuum evaporation crystallization techniques to enhance the MBR aqueous solubility with the purpose of overcoming the drawbacks of MBR and restraining the use of PEG 8000 during the drug formulation. Here, three water-soluble carboxylic acids (fumaric acid (FA, Figure 1b), l-pyroglutamic acid (PG, Figure 1c), and citric acid (CA, Figure 1d) whose pKa difference with MBR is greater than 3 were employed for co-amorphous formation.

This study aimed to report the intermolecular interactions occurring within MBR co-amorphouses (MBR-FA, MBR-PG, and MBR-CA) and to predict the interactions via solution-state nuclear magnetic resonance (NMR) (^1^H, ^13^C, and 2D) spectroscopy and attenuated total reflection Fourier transform infrared (ATR-FTIR) measurements. We also aimed to report comparative data regarding the solubilities, dissolution rates and phase transformation phenomena of MBR co-amorphouses, MBR crystalline, and MBR amorphous.

## 2. Materials and Methods

### 2.1. Materials

Mirabegron crystalline solid (purity 99.5%) was provided by Sungwun Pharmacopia Co., Ltd., (Seoul, Korea), APIs manufacturing company. Carboxylic acids including fumaric acid, l-pyroglutamic acid, and citric acid were purchased from Sigma-Aldrich (Darmstadt, Germany). Methanol was purchased form DaeJung Chem. Co., Ltd. (Siheung, Korea).

### 2.2. Mirabegron (MBR) Co-Amorphous Screening Using Vacuum Evaporation Crystallization

MBR has been reported to exhibit a poor solubility in organic solvents. Thus, a solubility test for MBR crystalline solid (50 mg) was conducted in distinct solvents such as methanol, ethanol, isopropyl alcohol, *n*-hexane, dichloromethane, and acetone (2 mL) to select a promising solvent. Methanol was selected as a result of this study because of its good ability to dissolve MBR crystalline solid. Afterwards, 20 g of MBR crystalline solid were measured and placed in each of the three 500 mL flasks. Then 1 equivalent (eq.) of carboxylic acids (FA: 5.9 g, PG: 6.5 g, CA: 9.7 g) (Figure 1b–d) were placed in each flask separately and dissolved with 300 mL methanol each. Vacuum evaporation crystallization technique was conducted on rotary evaporator (N-1300EVS Series, Eyela, Tokyo, Japan) (water base temperature, 50 °C and rotational speed, 200 rpm) to induce spontaneous formation of solid materials. Formed solid materials were dried at 25 °C for 8 h to produce MBR co-amorphouses.

### 2.3. Powder X-ray Diffraction (PXRD)

Powder X-ray diffraction (PXRD) analyses of MBR co-amorphouses were performed on Powder X-ray diffractometer (Bruker, D8 Advance, Billerica, MA, USA) using Cu Ka radiations. The voltage was 45 kV and the current was 40 mA. The divergence and scattering slits were set as 1°, while the receiving slit was 0.2 mm. Experiments were performed at a scan rate of 3°/min (0.4 s/0.02°) and 2θ scan ranged from 5° to 35°.

### 2.4. Solution-State Nuclear Magnetic Resonance (Solution-State NMR) Spectroscopy

1D (^1^H, ^13^C) and 2D (COSY, HMQC, and HMBC) analyses for MBR co-amorphouses (MBR-FA, MBR-PG, and MBR-CA), MBR crystalline solid, and carboxylic acids were performed on an 800 MHz high-resolution NMR Sspectrometer (Avance, Bruker, Billerica, MA, USA). DMSO-*d*_6_ was the solvent.

### 2.5. Solid-State Nuclear Magnetic Resonance (Solid-State CP/MAS ^13^C-NMR) Spectroscopy

The solid-state CP/MAS ^13^C-NMR measurements for MBR co-amorphouses (MBR-FA, MBR-PG, and MBR-CA), MBR crystalline solid, and carboxylic acids were recorded on 500 MHz solid-state NMR (Avance II, Bruker, Billerica, MA, USA) equipped with cross polarization (CP)/magic angle spinning (MAS) sequence pulse. Experimental condition was as follows: spinning, 5 KHz; pulse delay, 10 s; contact time, 2 min; analysis time, 24 h.

### 2.6. Attenuated Total Reflectance Fourier Transform Infrared Spectroscopy (ATR-FTIR)

ATR-FTIR spectroscopic analyses for MBR co-amorphouses (MBR-FA, MBR-PG, and MBR-CA), MBR crystalline solid, and carboxylic acids were carried out on an ATR-FTIR (PerkinElmer Spectrum 100 FT-IR) spectrometer equipped with a PerkinElmer Universal ATR Sampling Accessory (Massachusetts, Boston, MA, USA). The spectral range was set from 4000 to 650 cm^−1^ whereas the number of scans was 150.

### 2.7. Differential Scanning Calorimetry (DSC)

The thermal analyses of MBR co-amorphouses (MBR-FA, MBR-PG, and MBR-CA) and MBR amorphous were achieved on differential scanning calorimetry (DSC) equipment Q20 (TA Instruments, Philadelphia, PA, USA) instrument. The temperature ranged from 30 °C to 200 °C and the heating rate was set at 10 °C/min. The DSC measurements were conducted in nitrogen atmosphere using closed sample pans (Tzero pan and Lid, TA Instruments, Philadelphia, PA, USA). The DSC measurements were conducted in duplicate.

### 2.8. Dissolution Test of MBR Co-Amorphous

MBR co-amorphouses (MBR-FA, MBR-PG, MBR-CA), MBR crystalline and MBR amorphous powder materials were sieved (50 mesh) prior to the dissolution analysis for producing sample materials with uniform particle size. MBR amorphous samples of various concentrations including 10 μg/mL, 20 μg/mL, 30 μg/mL, 40 μg/mL, 50 μg/mL, 60 μg/mL and 70 μg/mL were prepared and calibrated via high-performance liquid chromatography (HPLC). The result shows an acceptable linearity of calibration with an *R*^2^ = 0.992. Afterwards, the dissolution rate investigations were conducted using an Agilent 708-DS Dissolution Apparatus (Santa Clara, CA, USA) at 37 °C. Sample materials for MBR co-amorphouses, MBR crystalline and MBR amosrphous were added separately (until precipitates were observed) in dissolution vessels containing 900 mL of water and then the mixtures were stirred at 100 rpm for 12 h. Later, 2 mL of each sample were withdrawn from each flask at 0 min, 10 min, 20 min, 25 min, 30 min, 35 min, 40 min, 50 min, 1 h, 2 h, 4 h, 6h, 8 h, 10 h and 12 h time intervals. Withdrawn supernatants were subsequently replaced with water of identical volume to maintain the dissolution condition. The collected supernatants were then diluted with MeOH and analyzed by HPLC (Agilent 1260, Santa Clara, CA, USA) to determine the concentrations. The HPLC analyses were performed using C_18_ column (4.6 × 150 mm, 5 μm, Kromasil^®^, Bohus, Sweden) and the mobile phase was A = 10 mM K_2_HPO_3_ water: methanol = 90:10 (*v*/*v*)/B = Acetonitrile:Methanol = 60:40 (*v*/*v*). The gradient elution condition of the mobile phase was: 0–8 min, 20–40% B; 8–15 min, 40–70% B; 15–20 min, 70% B; 20–25 min, 70–80% B; 25–30 min, 80% B. The flow rate was set as 1 mL/min, the UV detection wavelength as 250 nm, the injection volume as 5 μL and the run time as 30 min. The stationary phase temperature was kept at 30 °C. Furthermore, solid materials for MBR amorphous and MBR co-amorphouses of approximatively 30 mg were collected from the vessels bottom from 30 min of dissolution test for MBR amorphous and from 2 h of dissolution test for MBR co-amorphouses. The samples were thoroughly vacuum filtered (without further drying process) and were then analyzed by PXRD for monitoring their phase transformations.

## 3. Results and Discussion

### 3.1. Mirabegron Co-Amorphous Screening and Intermolecular Interactions

The screening of MBR co-amorphous was conducted using three carboxylic acids (FA, PG, and CA). These carboxylic acids were selected in regard with their water solubilities and on the basis of their pKa difference with MBR (pKa difference exceeding 3 (Table 1)).

**Figure 2:**
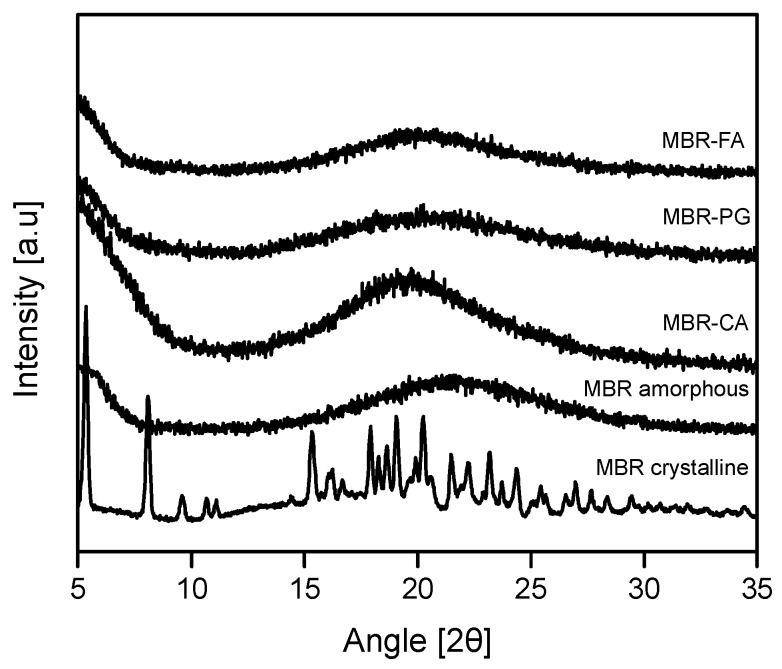
Powder X-ray diffraction (PXRD) patterns for MBR co-amorphouses (MBR-FA, MBR-PG, and MBR-CA), MBR amorphous and MBR crystalline.

One equivalent (eq.) of each carboxylic acid and MBR were separately dissolved in methanol which was selected from the solubility test and then mixed and crystallized by employing the vacuum evaporation crystallization technique. Afterwards, PXRD measurements were conducted to evaluate whether solid materials obtained were amorphous state solids. As a result, it was assessed that all MBR-FA, MBR-PG, and MBR-CA solids materials obtained in this study were amorphous state solids (Figure 2).

**Figure 3:**
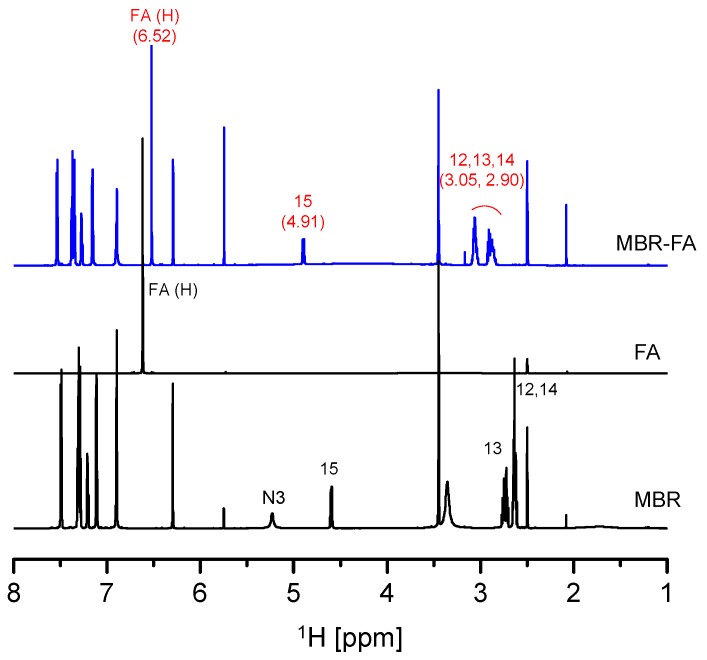
Solution-state nuclear magnetic resonance (^1^H-NMR) spectra for MBR-FA co-amorphous, MBR crystalline solid, and FA (numbers on peaks relate to numbers in Figure 1a structure and peak location) (DMSO-*d*_6_).

Solution-state NMR and FTIR spectroscopy are common analytical techniques employed to assess intermolecular interactions within solid materials with short-range order such as co-amorphous [15,16,17,18,19]. Figure 3 illustrates the solution-state ^1^H-NMR spectrum analysis result of MBR-FA co-amorphous solid. The analysis was performed for investigating the intermolecular interaction of MBR-FA co-amorphous. From the results, it can be observed that proton shifts of H atom related to C12, C13, C14, and C15 (Figure 1a) downfield movement in MBR-FA spectrum compared to MBR spectrum. Moreover, peaks related to the FA molecule were observed to shift upfield in MBR-FA spectrum than in FA spectrum. These results are attributed to the decrease of C12, C13, C14, and C15 electron distribution resulting from the protonation of MBR’s N3 group (Figure 1a) and the increase of FA’s H electron distribution resulting from the deprotonation of the carboxylic acid group of FA during the formation process of MBR-FA co-amorphous (Figure 3). Besides, the solution-state ^13^C-NMR spectra displays similarity with the solution-state ^1^H-NMR spectra in Figure 3 in terms of characteristic peaks’ chemical shift in Figure S1. Accordingly, it was confirmed that MBR and FA molecules are linked via ionic interactions to form MBR-FA co-amorphous.

**Figure 4:**
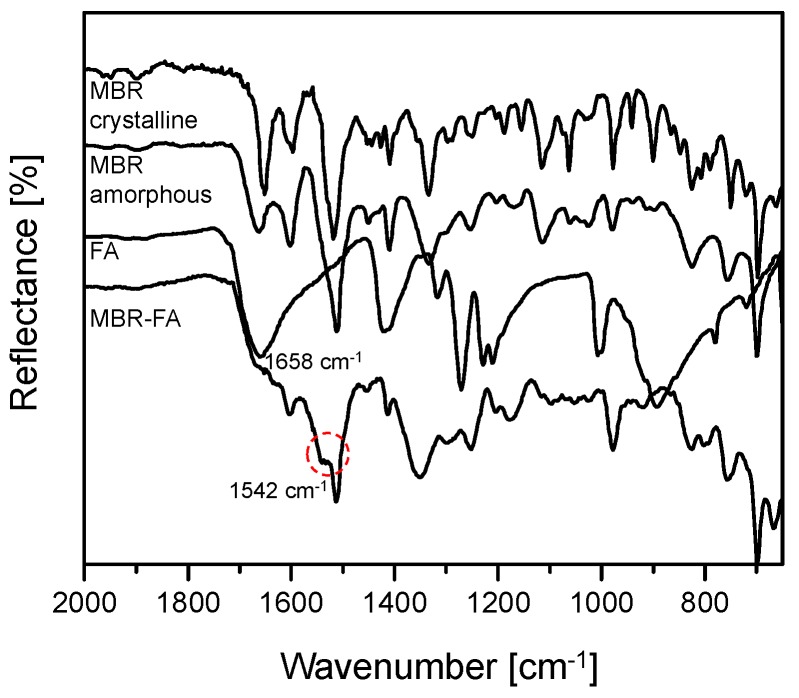
ATR-FTIR spectra for MBR-FA co-amorphous, MBR amorphous, MBR crystalline solid, and FA.

Yamamura et al. [15] suggested that during co-amorphous formation, carboxylic acid undergoes deprotonation and converts to carboxylate ion. In this case, the C=O absorption peak of carboxylic acid is observed to shift from 1650 cm^−1^ to 1580 cm^−1^. In the ATR FTIR spectra illustrated in the Figure 4, it can be observed that the C=O absorption peak of single FA appeared at 1658 cm^−1^ whereas the peak assumed as C=O absorption peak of FA in MBR-FA molecule appeared at 1542 cm^−1^. The present FTIR spectra display tight similarity with the FT-IR spectra suggested by Yamamura et al. [15]. From the spectral analysis of FTIR results in Figure 4, we could confirm that FA carboxylic acid was deprotonated to form carboxylate ion during co-amorphous formation. Therefore, it was predicted that MBR and FA molecules interact via ionic interaction to produce MBR-FA co-amorphous.

MBR-PG and MBR-CA co-amorphouses solution-state NMR spectra display similar chemical shifts with Figure 3 and Figure S1 spectra for C12, C13, C14, and C15’s H peaks (Figure 1a) and carboxylic acids’ H peaks. Thus, it was assumed that both MBR-PG and MBR-CA co-amorphouses were formed through the ionic interactions between MBR and carboxylic acids, which result from the protonation of MBR’s N3 group (Figure 1a) and the deprotonation of carboxylic acid group. Moreover, as can be seen in MBR-PG and MBR-CA co-amorphouses FTIR spectra, it can be noticed that the C=O absorption peak of PG in the single PG molecule appeared at 1636 cm^−1^ while the peak assumed as C=O absorption peak of PG in MBR-PG co-amorphous appeared at 1539 cm^−1^. In the same FTIR data, it can be observed that the C=O absorption peak of CA in the single CA molecule appeared at 1645 cm^−1^ whereas the peak assumed as C=O absorption peak of CA in MBR-CA co-amorphous molecule appeared at 1544 cm^−1^. These FTIR data revealed similar patterns with FTIR spectra suggested by Yamamura et al. [15]. Like FTIR data result in Figure 4, it was predicted that both PG and CA carboxylic acids undergo deprotonation and thereby convert to carboxylate anions during the formation of co-amorphouses (Figures S2–S7). These results suggested that all MBR co-amorphouses obtained in this study were produced via ionic interaction between MBR and carboxylic acids to form short-range order materials namely amorphous state solids. The solution-state ^1^H- and ^13^C-NMR spectra of MBR co-amorphouses were interpreted and assessed via the solution-state 2D NMR (^1^H-^1^H COSY, ^1^H-^13^C HSQC, and ^1^H-^13^C HMBC) analysis results (Figures S8–S19). Moreover, the integrated solution-state ^1^H-NMR spectra of co-amorphouses demonstrated that MBR and carboxylic acids interact in 1:1 ratio to produce MBR-FA, MBR-PG, and MBR-CA co-amorphouses (Figures S20–S22).

**Figure 5:**
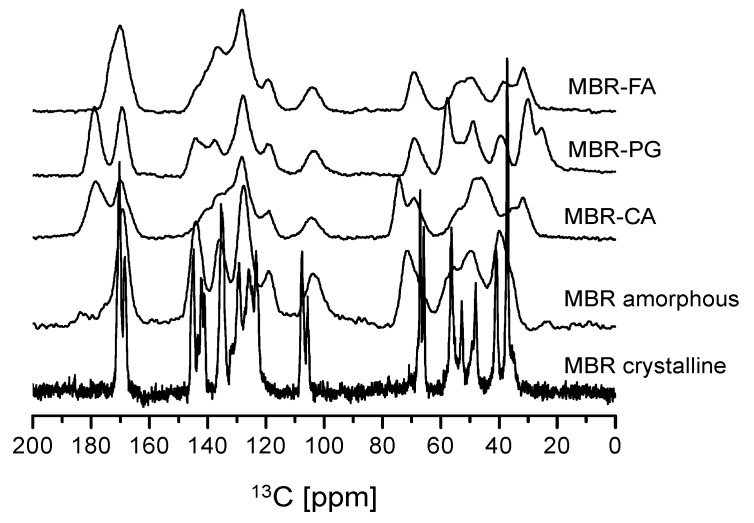
Solid-state CP/MAS ^13^C-NMR spectra of MBR co-amorphouses (MBR-FA, MBR-PG, and MBR-CA), MBR amorphous and MBR crystalline solid.

Amorphous materials are characterized by the presence of broad and fused peaks without splitting their solid-state CP/MAS ^13^C-NMR spectra when compared to crystalline materials [31,32]. Figure 5 illustrates the comparison of solid-state CP/MAS ^13^C-NMR spectra of MBR co-amorphouses (MBR-FA, MBR-PG, and MBR-CA) and MBR crystalline. In Figure 5, all the MBR co-amorphouses are clearly distinctive when taking the difference in their spectral peaks into an account. When compared to the spectrum of MBR crystalline, the co-amorphous solids possess broad and fused peaks in their spectra. From the interpretation of solid-state CP/MAS ^13^C-NMR spectra in Figure 5, MBR-FA, MBR-PG, and MBR-CA co-amorphouses were assessed to be amorphous state materials. However, the presence of broader and fused co-amorphous peaks hinder the further and detailed spectral interpretation of MBR co-amorphouses (Figure 5). A comparative evaluation of solid-state CP/MAS ^13^C-NMR spectra of MBR co-amorphouses with single MBR and carboxylic acids was conducted to deduce and confirm a clear difference among peaks of MBR-FA, MBR-PG, and MBR-CA co-amorphouses (Figures S23–S25).

### 3.2. Dissolution Test for MBR Co-Amorphous and Investigation of Their Phase Transformation to Crystalline Solid

**Figure 6:**
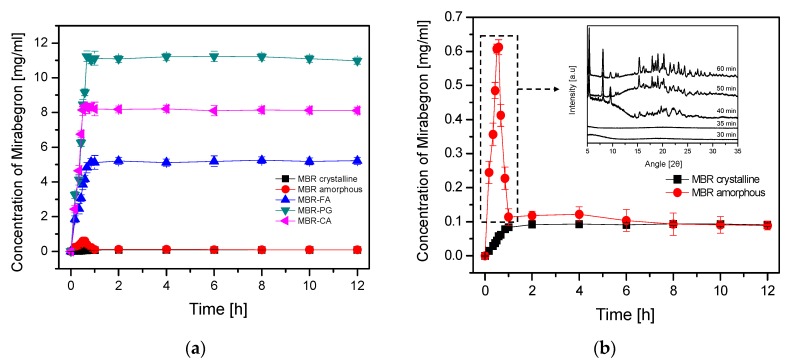
Dissolution profiles for MBR co-amorphouses, MBR crystalline solid, and MBR amorphous; (**a**) dissolution profiles of MBR co-amorphouses (MBR-FA, MBR-PG, and MBR-CA), MBR crystalline solid, and MBR amorphous at 37 °C; (**b**) dissolution profiles of MBR crystalline and MBR amorphous at 37 °C and monitoring of MBR amorphous phase transformation using PXRD.

**Figure 7:**
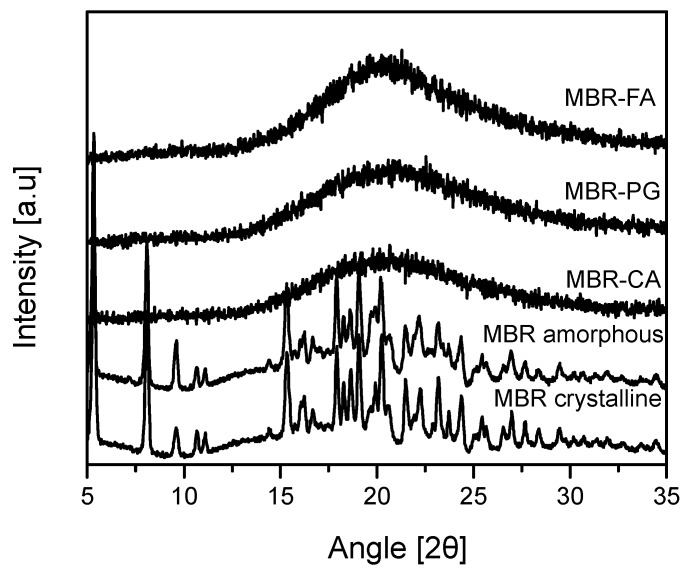
PXRD of MBR solids collected after 12 h of dissolution test.

Powdered MBR-FA, MBR-PG and MBR-CA co-amorphouses, MBR crystalline and MBR amorphous (50 mesh) were analyzed for evaluating their solubilities as well as their dissolution rates. For dissolution rate investigations, all sample materials were individually transferred in a dissolution tester containing 900 mL water at 37 °C. Sample materials were added until precipitates were observed and then the mixtures were stirred at 100 rpm for 12 h. Afterwards, 2 mL of supernatants as well as approximatively 30 mg of solid materials of each sample were withdrawn from each dissolution vessel at different sample time intervals. Water of identical volume with withdrawn supernatants was subsequently added to each dissolution vessel to maintain the dissolution condition. The withdrawn supernatants were analyzed via HPLC to determine the concentrations (Figures S26–S28) whereas the solid materials were monitored via PXRD to investigate the occurrence of the phase transformation during the dissolution process. In Figure 6 and Figure 7 are depicted the dissolution profiles and PXRD phase transformation monitoring results for MBR co-amorphouses, MBR crystalline solid, and MBR amorphous. The dissolution profiles of MBR co-amorphouses, MBR crystalline solid, and MBR amorphous illustrated in Figure 6a revealed that the equilibrium concentrations were 5.15 mg/mL for MBR-FA co-amorphous, 11.12 mg/mL for MBR-PG co-amorphous and 8.15 mg/mL for MBR-CA co-amorphous, respectively. The equilibrium concentrations of MBR-FA, MBR-PG, and MBR-CA co-amorphouses were found to be respectively 57-fold, 123-fold, and 90-fold greater than MBR crystalline solid (0.09 mg/mL). Besides, in Figure 6b, the equilibrium concentration of MBR amorphous was 0.61 mg/mL which was found to be 6.7-fold higher than that of MBR crystalline solid. Therefore, from the results, the increased solubility and dissolution rate of MBR amorphous was assessed. However, through dissolutions and PXRD phase transformation monitoring results of MBR crystalline solid and MBR amorphous, illustrated in Figure 6b, it could be noticed that MBR amorphous start converting to crystalline after 35 min, and within 1 h all MBR amorphous convert to crystalline form. Based on these results, MBR amorphous was considered as an inadequate API substance for drug development due to its extremely weak thermodynamic stability which leads to its rapid phase transformation into crystalline solids.

Nevertheless, unlike MBR amorphous (Figure 6b), no change in the equilibrium concentration could be observed for MBR co-amorphouses (Figure 6a). In addition, from the PXRD monitoring test, no conversion of MBR co-amorphouses to crystalline form was observed during the 12 h of dissolution test (Figure 7).

Accordingly, we could assess that MBR co-amorphouses possess a remarkably greater thermodynamic stability compared to MBR amorphous as no phase transformation of these co-amorphous materials to crystalline form was observed within the 12 h dissolution process. The ionization of both MBR and carboxylic acid molecules followed by the formation of amorphous state materials via ionic interaction between MBR and carboxylic acid ions are considered the main reason leading to the production of MBR co-amorphouses possessing increased solubilities and dissolution rates compared to MBR crystalline solid and MBR amorphous. Therefore, MBR co-amorphouses have been assessed as potential API which can overcome the drawback of MBR crystalline solid, especially its aqueous solubility.

**Figure 8:**
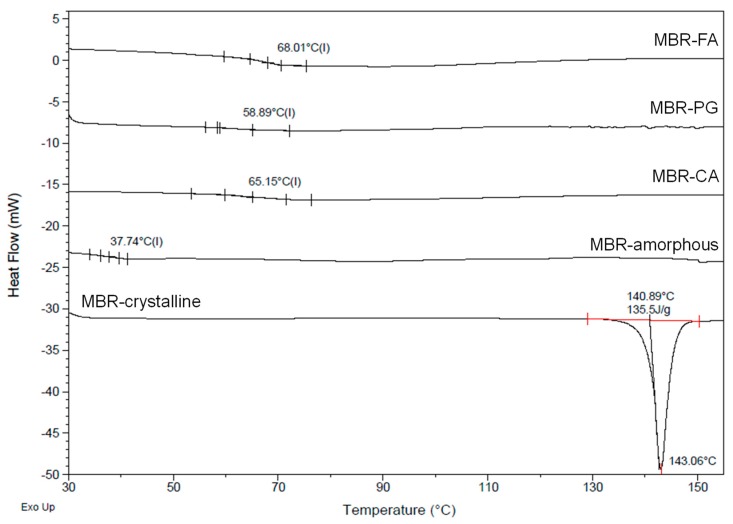
Differential scanning calorimetry (DSC) curves of MBR co-amorphouses (MBR-FA, MBR-PG, and MBR-CA), MBR crystalline, and MBR amorphous (heating rate 10 °C /min).

The high *T_g_* values of amorphous materials influence their physicochemical stabilities, inhibit the process leading to their conversion into crystalline solids and, consequently, promote the storage stability of amorphous state drugs during a long period [4,5,6,7,14]. Additionally, co-amorphous materials formed via ionic interaction between different molecules are assumed to possess high *T_g_* values compared to the amorphous API itself [33]. Figure 8 presents DSC measurements (curves) collected for the evaluation and comparison of MBR co-amorphouses and MBR amorphous’ *T_g_* values. From the results, the *T_g_* value of MBR amorphous could be observed at approximatively 37.74 °C whereas the *T_g_* values of MBR co-amorphouses appeared above 58 °C. From this result, we were able to predict that MBR co-amorphouses (Figure 6 and Figure 7) were more thermodynamically stable than MBR amorphous. The high *T_g_* values observed for MBR co-amorphouses were assumed to slow the phase transformation process of these solid materials to crystalline form and to promote their amorphic stability for the 12 h dissolution test (Figure 6). In other words, the presence of ionic interactions within MBR co-amorphouses promotes an increase in *T_g_* value of each co-amorphous, which leads to an increase in their stability and consequently to prevention of the phase transformation to crystalline materials (even after 12 h).

When comparing the glass transition temperatures (*T_g_*) of MBR co-amorphouses (MBR-FA, MBR-PG, and MBR-CA) in Figure 8, we can notice that their stabilities decrease in the following order; MBR-FA > MBR-CA > MBR-PG. The difference in the strength of the ionic interaction between MBR and carboxylic acids is assumed to be the main factor influencing the stability of MBR co-amorphouses. According to Table 1, the pKa values of these carboxylic acids decrease as follows; FA (3.03) > CA (3.13) > PG (3.32). The acidity of FA is assumed to be 2-fold and 1.26-fold stronger than PG and CA, respectively [30]. Moreover, CA is assumed to be 1.58-fold more highly acidic than PG. In this regard, the difference in stability of MBR-co-amorphous, depending on the *T_g_* values (Figure 8), is essentially attributed to the difference in acidity of the carboxylic acids. The acidity is assumed to impact the strength of ionic interaction between MBR and carboxylic acids and, therefore, influence the stability of the resulting co-amorphous materials. Because of its high acidic level compared to other carboxylic acids, FA binds to MBR through a strong ionic interaction. This increases the *T_g_* of the material formed and leads to the formation of highly stable MBR-FA co-amorphous. Besides, CA possesses intermediate acidity, lower than that of FA but higher than that of PG. Therefore, it is assumed that CA bonds to MBR molecule via ionic interaction of intermediate strength. This consequently leads to the formation of MBR-CA co-amorphous of intermediate *T_g_* and intermediate stability. Lastly, MBR-PG co-amorphous is considered the least stable among others due to the inferior PG’s acidic property and the weak ionic interaction between PG and MBR in MBR-PG co-amorphous.

However, the results for the dissolution investigation for MBR co-amorphouses illustrated in Figure 6a demonstrate a completely different tendency to the stability results depicts in Figure 8. In Figure 6a, the solubilities of MBR co-amorphouses are as follows; MBR-PG > MBR-CA > MBR-FA. Here, the difference in the dissolution properties of MBR-co-amorphouses is assumed to be the consequence of the difference in acidic levels (pKa) between MBR and carboxylic acids (Table 1). According to the result, MBR-FA co-amorphous displays a very low solubility compared to other co-amorphouses. This is because of the high acidic difference and strong ionic interaction between MBR and FA and because of the comparatively high stability of the co-amorphous material. Besides, MBR-CA co-amorphous are assumed to exhibit moderate solubility because of the presence of average ionic force between MBR and CA in the co-amorphous molecule and because of the material’s moderated stability. Lastly, MBR-PG co-amorphous exhibits great solubility because of the feeble ionic interaction between MBR and PG in MBR-PG co-amorphous and the poor stability of the material. 

Collectively, the results demonstrated that the obtained MBR co-amorphouses (MBR-FA, MBR-PG, and MBR-CA) possess the increased solubilities and dissolution rates of above 57-fold to the MBR crystalline. The same results revealed that MBR co-amorphouses were remarkably stable and could maintain their amorphous state for more than 12 h. In this regard, MBR co-amorphouses are assumed as promising API capable to overcome the solubility problem of MBR crystalline solid and to improve MBR drugs’ adsorption rates in the body even without the employment of PEG 8000 hydrophilic base during the formulation process.

Nevertheless, data specifying the phase transition time from MBR co-amorphouses to crystalline form and investigating their salt forms (MBR-FA, MBR-PG, and MBR-CA) were not reported in the present study. Thus, our future plan is to investigate the phase transformation phenomenon of MBR-FA, MBR-PG, and MBR-CA co-amorphouses and to screen and characterize their salt forms and polymorphs.

## 4. Conclusions

In this work, the screening of MBR co-amorphouses was carried out with the purpose of enhancing the aqueous solubility of MBR crystalline solid, improving its absorption, and thus limiting the use of PEG 8000, a harmful hydrophilic base, employed during MBR drug formulation. As a result, MBR co-amorphouses were produced when FA, PG, and CA carboxylic acids were employed as coformers. Moreover, by means of PXRD, solution-state NMR, ATR-FTIR, and solid-state CP/MAS ^13^C-NMR analyses, it was assessed that MBR and carboxylic acids molecules were linked together via ionic interactions to form MBR co-amorphouses. In addition, the results for a 12 h dissolution test of MBR co-amorphouses, MBR crystalline solid, and MBR amorphous demonstrated that the aqueous solubilities as well as the dissolution rates of MBR co-amorphouses improved more than 57-fold compared to MBR crystalline solid. The same results revealed that the solubility and dissolution rate of MBR amorphous increased approximatively 6.7-fold compared to MBR crystalline solid. However, from the PXRD monitoring result it was noticed that the phase transformation of MBR amorphous to crystalline solid commences in approximatively 35 min and can be completed within an hour. Therefore, MBR amorphous was assumed to be a thermodynamically unstable solid. Nevertheless, MBR co-amorphouses were assessed to be thermodynamically stable solids as no conversion of these co-amorphouses to crystalline solids could be observed during the 12 h of dissolution test. The increase of MBR co-amorphouses’ stabilities have been attributed to the presence of ionic interactions within MBR co-amorphous molecules, which elevate the molecules’ *T_g_* values, promote their stabilities and, consequently, prevent their phase transformation to crystalline solids.

MBR co-amorphouses including MBR-FA, MBR-PG, and MBR-CA obtained in this work, display enhanced aqueous solubilities owing the occurrence of ionic interactions within these molecules. Unlike MBR amorphous, the presence of ionic interactions within MBR co-amorphouses is presumed to promote the *T_g_* values of MBR co-amorphouses, improve their thermodynamic stabilities and, subsequently, slow down their conversion processes to crystalline solids. From this study, it was assessed that MBR co-amorphouses were potential APIs, capable of overwhelming the limitations of MBR crystalline solid because of their improved properties including aqueous solubility and thermodynamic stability. In this regard, we foresee that MBR co-amorphous alone can exhibit higher absorption rate even without using the PEG 8000 hydrophilic base during drug formulation.

## Figures and Tables

**Table 1:** pKa values of carboxylic acids employed for the screening of Mirabegron (MBR) co-amorphous (MBR pKa 8).

Carboxylic Acid	pKa (Ref [30])
Fumaric acid (FA) (Figure 1b)	(1) 3.03, (2) 4.38
l-Pyroglutamic acid (PG) (Figure 1c)	3.32
Citric acid (CA) (Figure 1d)	(1) 3.13, (2) 4.76, (3) 6.40

## Supplementary Materials

The following are available online at http://www.mdpi.com/1999-4923/10/3/149/s1, Figure S1: Solution-state ^13^C-NMR spectra for MBR-FA co-amorphous, MBR and FA (DMSO-*d*_6_); Figure S2: Solution-state ^1^H-NMR spectra for MBR-PG co-amorphous, MBR, and PG (DMSO-*d*_6_); Figure S3: Solution-state ^13^C-NMR spectra for MBR-PG co-amorphous and MBR (DMSO-*d*_6_); Figure S4: ATR-FTIR spectra for MBR-PG co-amorphous, MBR, and PG; Figure S5: Solution-state ^1^H-NMR spectra for MBR-CA co-amorphous, MBR, and CA (DMSO-*d*_6_); Figure S6: Solution-state ^13^C-NMR spectra for MBR-CA co-amorphous, MBR, and CA (DMSO-*d*_6_); Figure S7: ATR-FTIR spectra for MBR-CA co-amorphous, MBR, and CA; Figure S8: Solution-state 2D (^1^H-^1^H COSY) NMR spectrum of MBR (DMSO-*d*_6_); Figure S9: Solution-state 2D (^1^H-^13^C HSQC) NMR spectrum of MBR (DMSO-*d*_6_); Figure S10: Solution-state 2D (^1^H-^13^C HMBC) NMR spectrum of MBR (DMSO-*d*_6_); Figure S11: Solution-state 2D (^1^H-^1^H COSY) NMR spectrum of MBR-FA co-amorphous (DMSO-*d*_6_); Figure S12: Solution-state 2D (^1^H-^13^C HSQC) NMR spectrum of MBR-FA co-amorphous (DMSO-*d*_6_); Figure S13: Solution-state 2D (^1^H-^13^C HMBC) NMR spectrum of MBR-FA co-amorphous (DMSO-*d*_6_); Figure S14: Solution-state 2D (^1^H-^1^H COSY) NMR spectrum of MBR-PG co-amorphous (DMSO-*d*_6_); Figure S15: Solution-state 2D (^1^H-^13^C HSQC) NMR spectrum of MBR-PG co-amorphous (DMSO-*d*_6_); Figure S16: Solution-state 2D (^1^H-^13^C HMBC) NMR data of MBR-PG co-amorphous (DMSO-*d*_6_); Figure S17: Solution-state 2D (^1^H-^1^H COSY) NMR spectrum of MBR-CA co-amorphous (DMSO-*d*_6_); Figure S18: Solution-state 2D (^1^H-^13^C HSQC) NMR spectrum of MBR-CA co-amorphous (DMSO-*d*_6_); Figure S19: Solution-state 2D (^1^H-^13^C HMBC) NMR spectrum of MBR-CA co-amorphous (DMSO-*d*_6_); Figure S20: Solution-state ^1^H-NMR spectrum of MBR-FA co-amorphous; Figure S21: Solution-state ^1^H-NMR spectrum of MBR-PG co-amorphous; Figure S22: Solution-state ^1^H-NMR spectrum of MBR-CA co-amorphous; Figure S23: Solid-state CP/MAS ^13^C-NMR spectra for MBR-FA co-amorphous, MBR, and FA; Figure S24: Solid-state CP/MAS ^13^C-NMR spectra for MBR-PG co-amorphous, MBR, and PG; Figure S25: Solid-state CP/MAS ^13^C-NMR spectra for MBR-CA co-amorphous, MBR, and CA; Figure S26: HPLC chromatograms of the dissolution of MBR-FA at 10 min, 20 min, 60 min and 720 min; Figure S27: HPLC chromatograms of the dissolution of MBR-PG at 10 min, 20 min, 60 min and 720 min; Figure S28: HPLC chromatograms of the dissolution of MBR-CA at 10 min, 20 min, 60 min and 720 min.
