# Supplementary material for: Co-Amorphous Screening for the Solubility Enhancement of Poorly Water-Soluble Mirabegron and Investigation of Their Intermolecular Interactions and Dissolution Behaviors
Source: Pharmaceutics. 2018 Sep 5;10(3):149. doi: 10.3390/pharmaceutics10030149 (PMC6161252; doi:10.3390/pharmaceutics10030149)
Supplement: Supplementary file 1 [file pharmaceutics-10-00149-s001.pdf]

pharmaceutics

MDPI

Supplementary Materials: Co-Amorphous Screening for the Solubility Enhancement of Poorly Water-Soluble Mirabegron and Investigation of Their Intermolecular Interactions and Dissolution Behaviors

Ji-Hun An, Changjin Lim, Alice Nguvoko Kiyonga, In Hwa Chung, In Kyu Lee, Kilwoong Mo, Minho Park, Wonno Youn, Won Rak Choi, Young-Ger Suh and Kiwon Jung

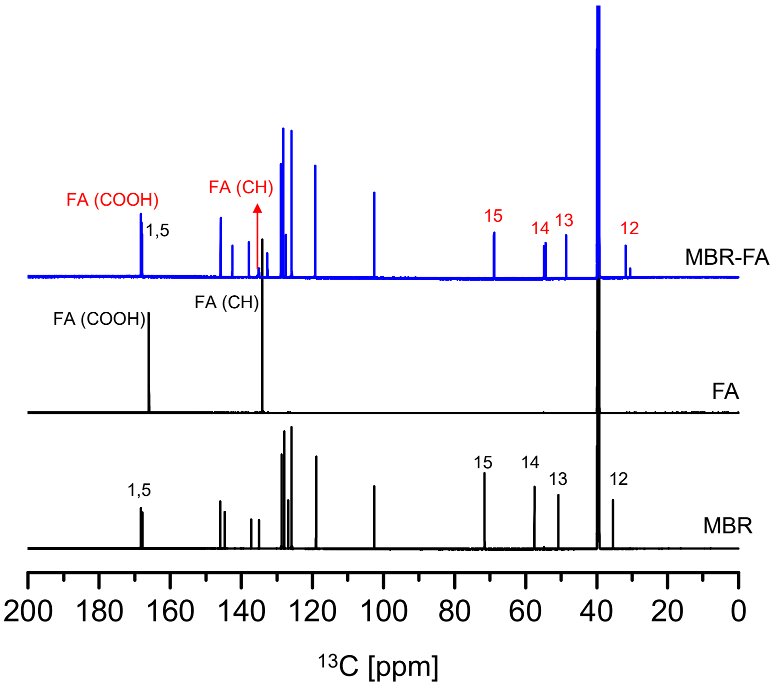

Figure S1. Solution-state ^{13}C-NMR spectra for MBR-FA co-amorphous, MBR and FA (DMSO-d_6).

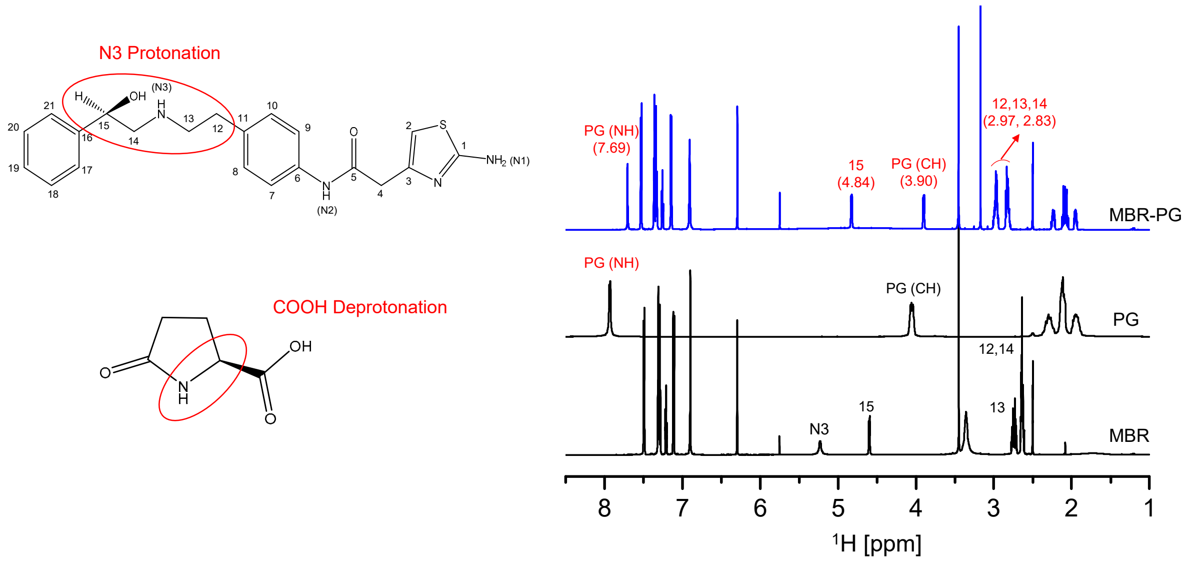

Figure S2. Solution-state ^{1}H-NMR spectra for MBR-PG co-amorphous, MBR and PG (DMSO-d_6).

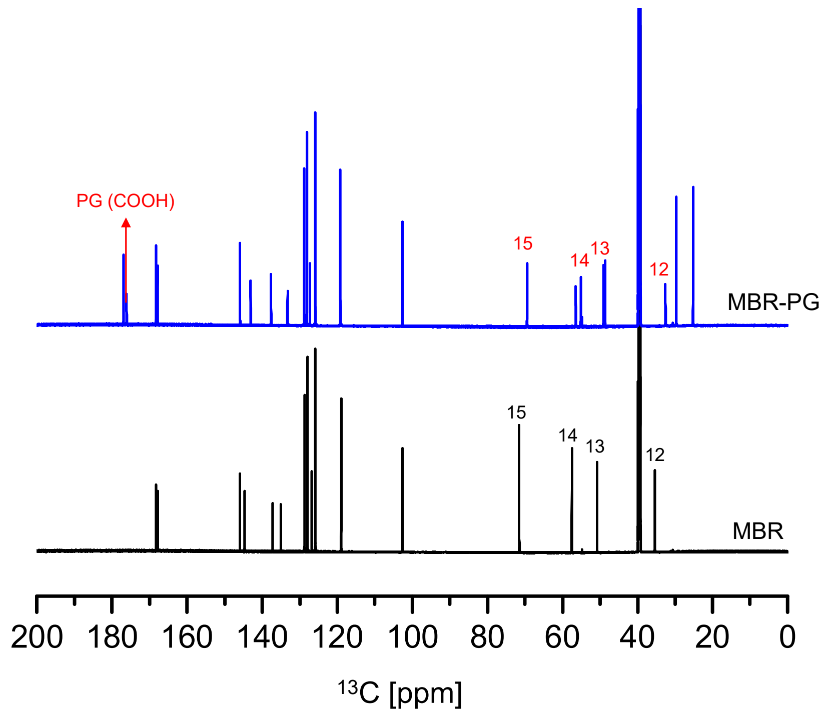

Figure S3. Solution-state ^{13}C-NMR spectra for MBR-PG co-amorphous and MBR (DMSO-d_6).

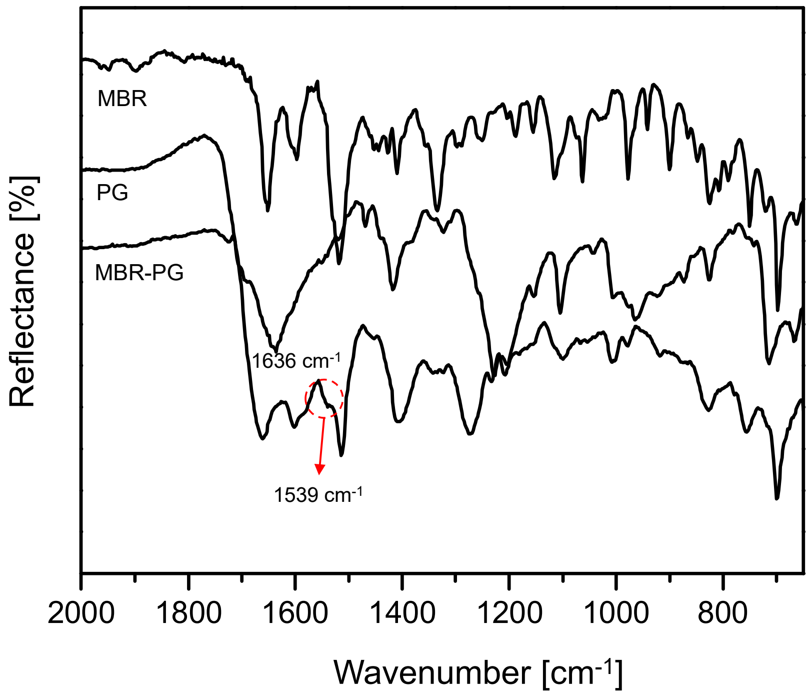

Figure S4. ATR-FTIR spectra for MBR-PG co-amorphous, MBR and PG.

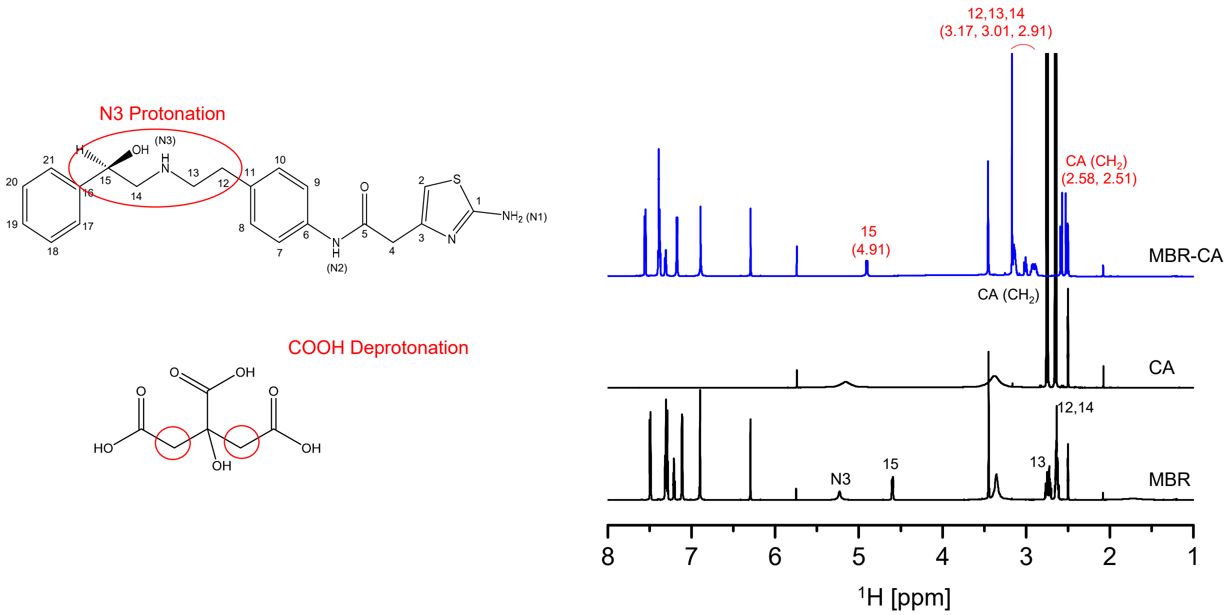

Figure S5. Solution-state ^{1}H-NMR spectra for MBR-CA co-amorphous, MBR and CA (DMSO-d_6).

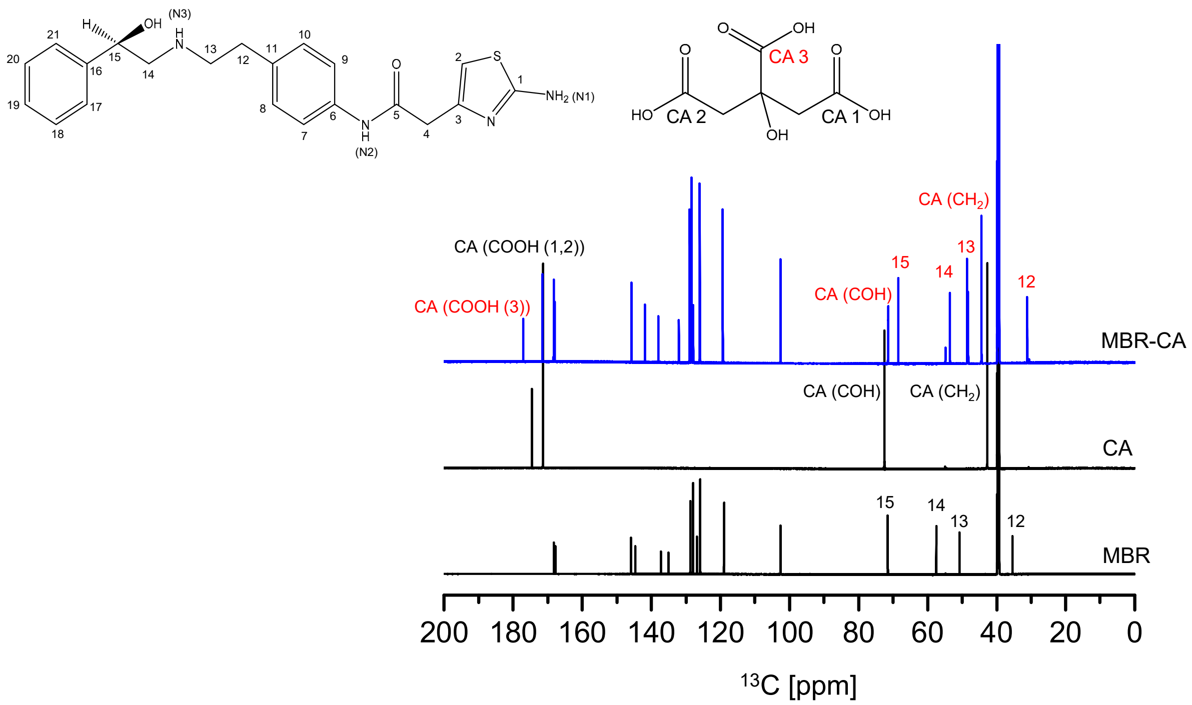

Figure S6. Solution-state ^{13}C-NMR spectra for MBR-CA co-amorphous, MBR and CA (DMSO-d_6).

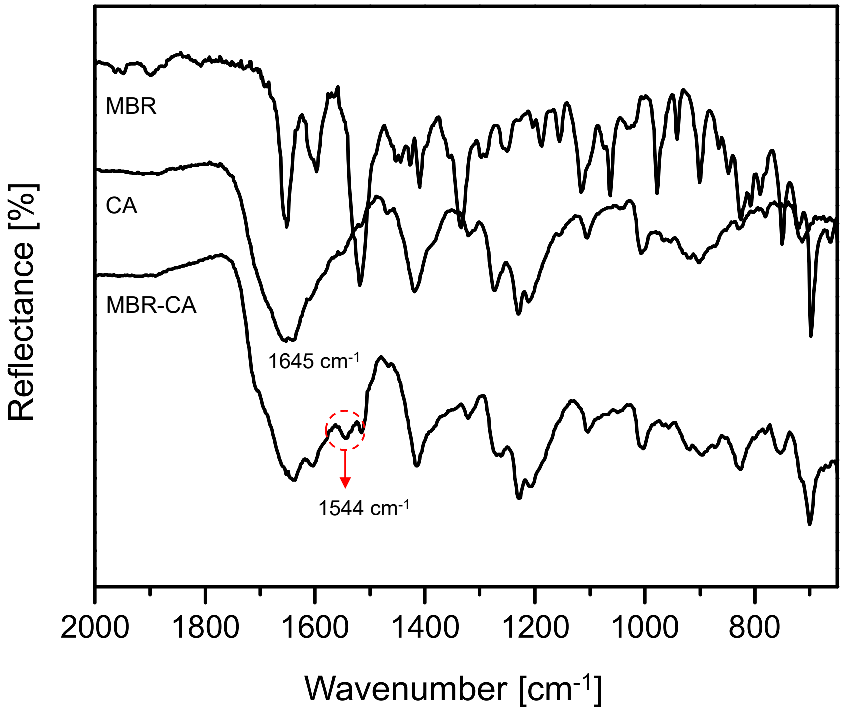

Figure S7. ATR-FTIR spectra for MBR-CA co-amorphous, MBR and CA.

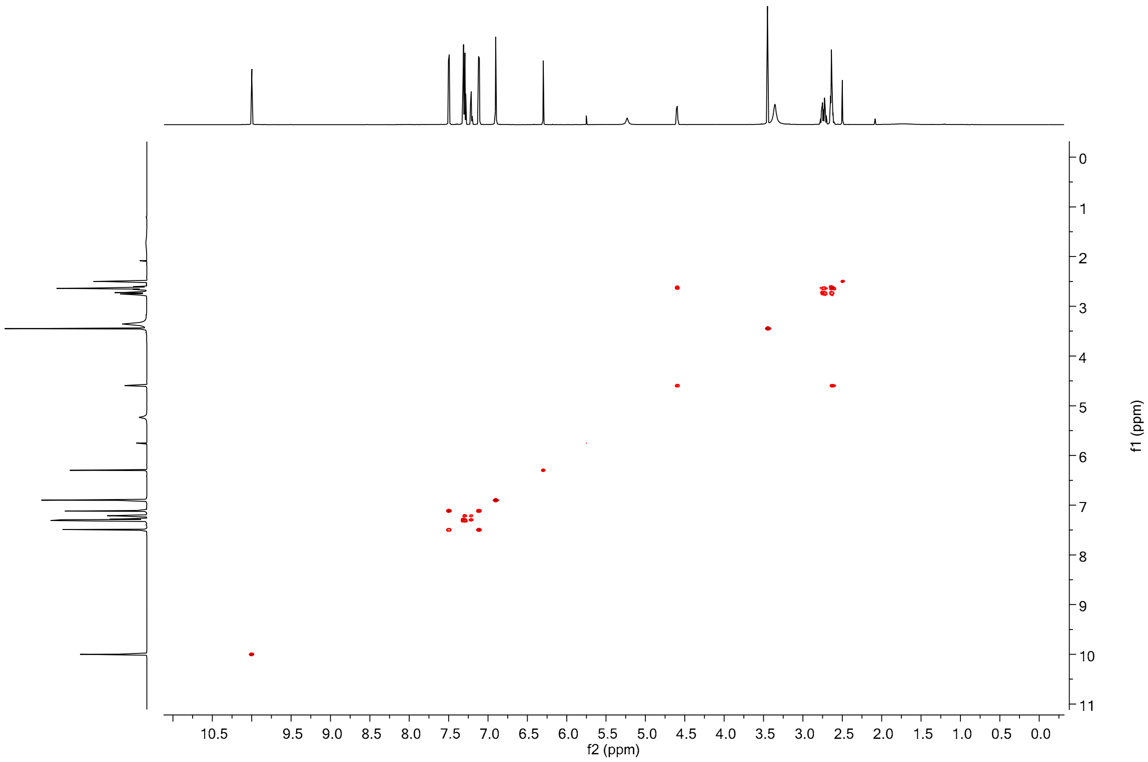

Figure S8. Solution-state 2D (^{1}H-^{1}H COSY) NMR spectrum of MBR (DMSO-d_6).

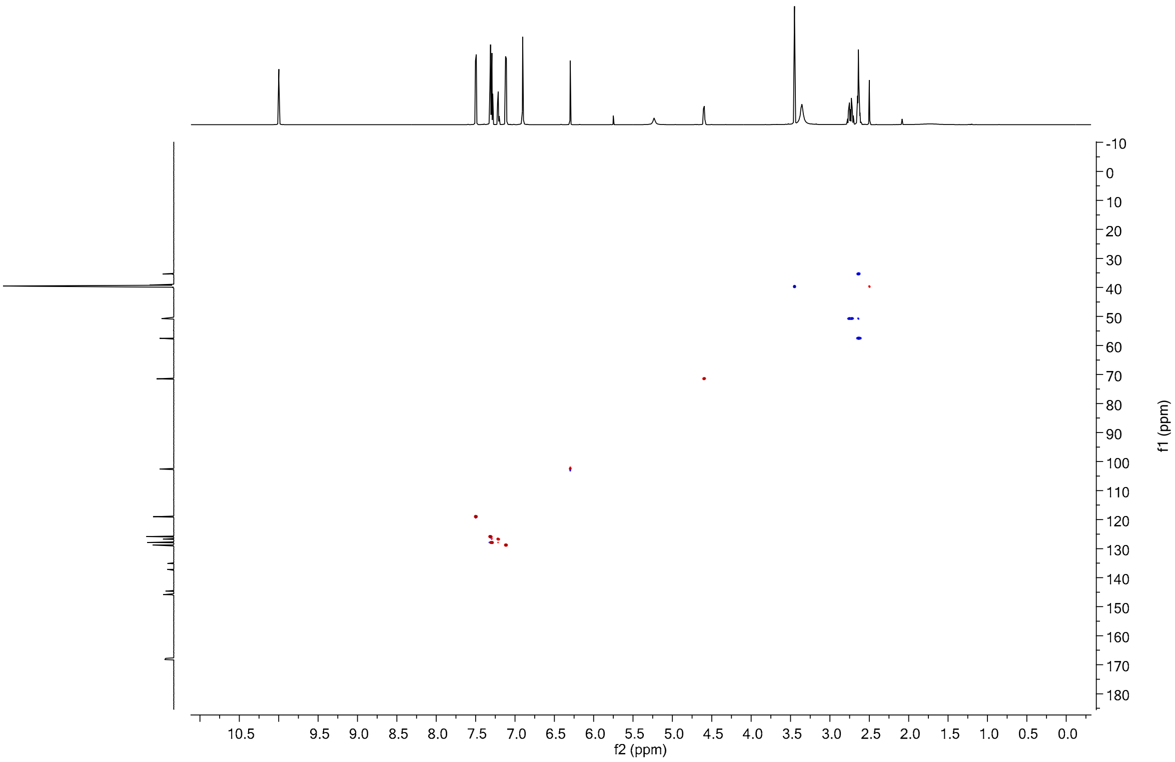

Figure S9. Solution-state 2D (^{1}H-^{13}H HSQC) NMR spectrum of MBR (DMSO-d_6).

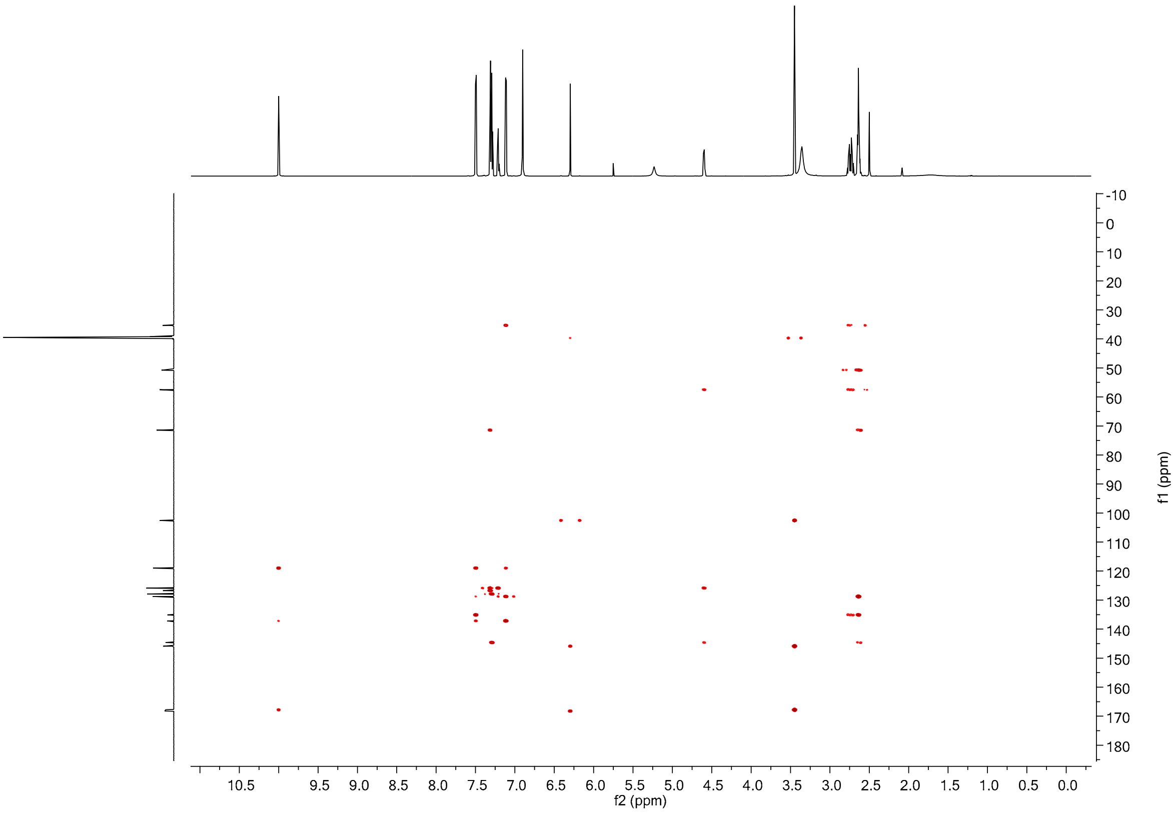

Figure S10. Solution-state 2D (^{1}H-^{13}H HMBC) NMR spectrum of MBR (DMSO-d_6).

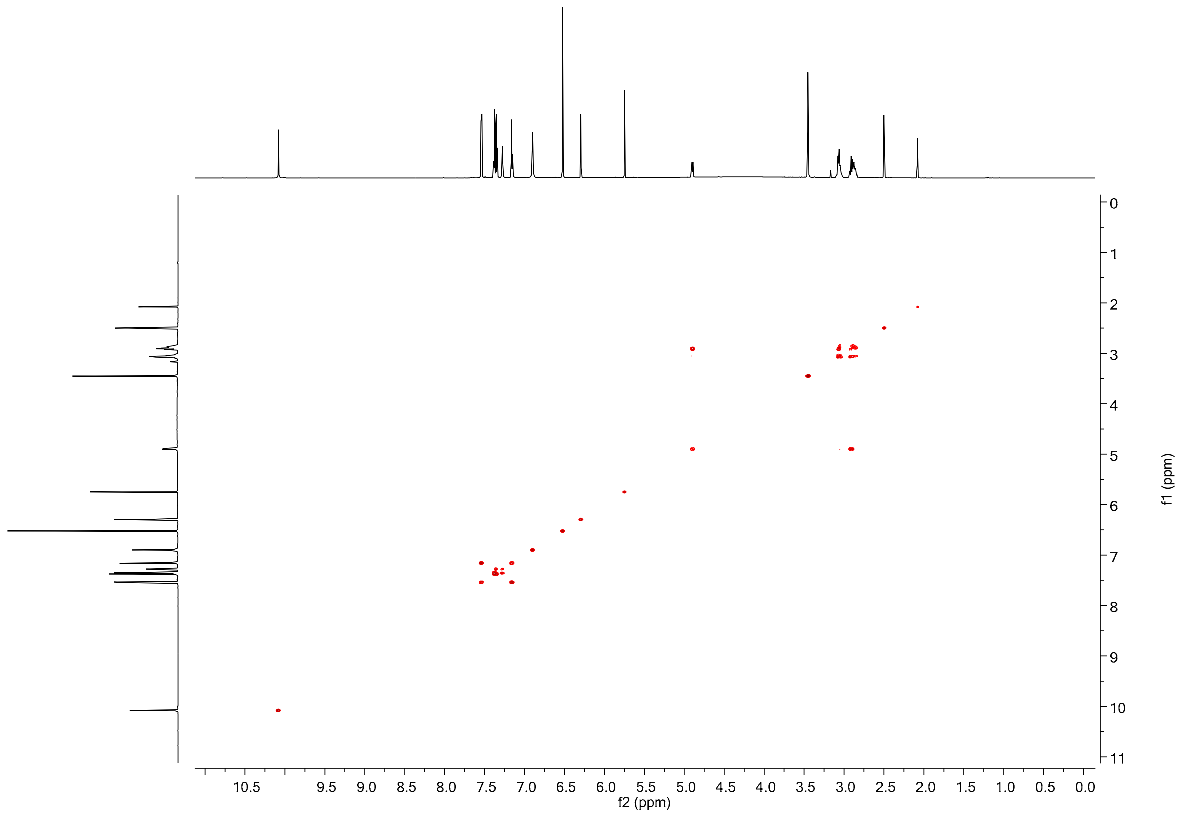

Figure S11. Solution-state 2D (^{1}H-^{1}H COSY) NMR spectrum of MBR-FA co-amorphous (DMSO-d_6).

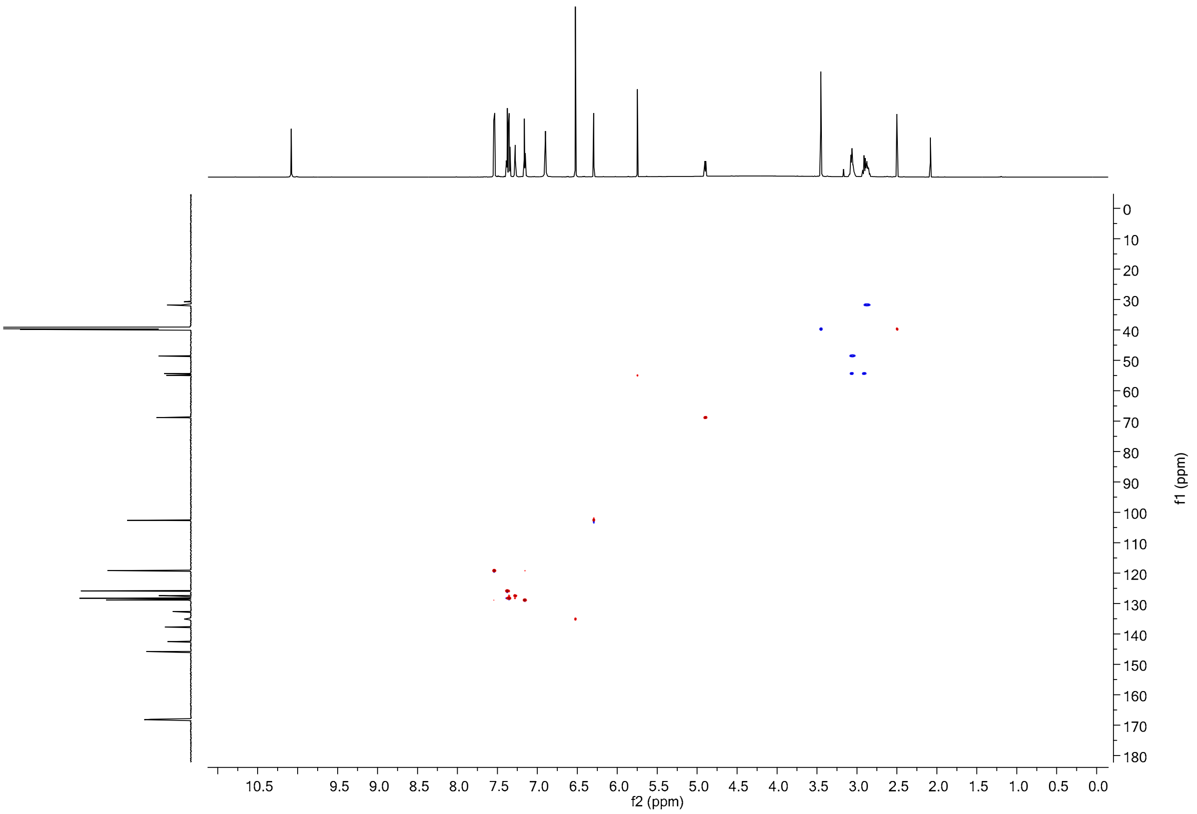

Figure S12. Solution-state 2D (^{1}H-^{13}C HSQC) NMR spectrum of MBR-FA co-amorphous (DMSO-d_6).

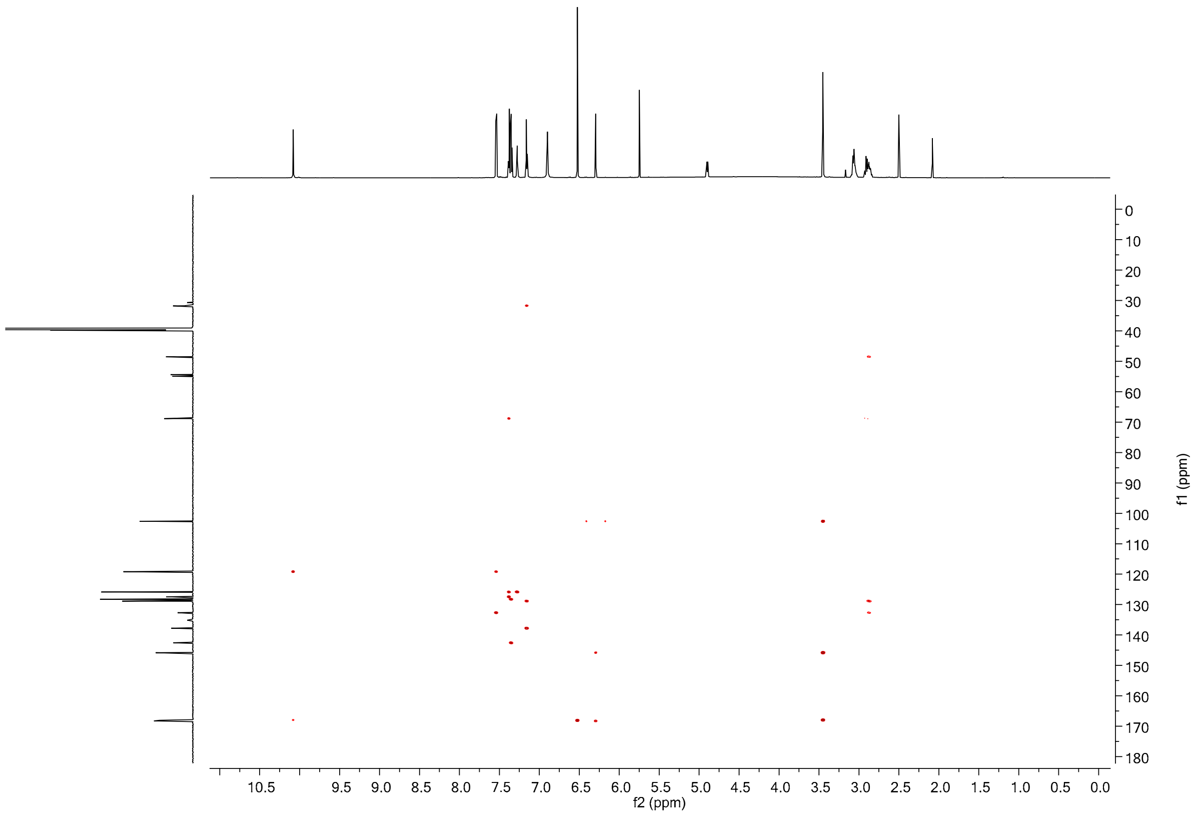

Figure S13. Solution-state 2D (^1H-^{13}C HMBC) NMR spectrum of MBR-FA co-amorphous (DMSO-d_6).

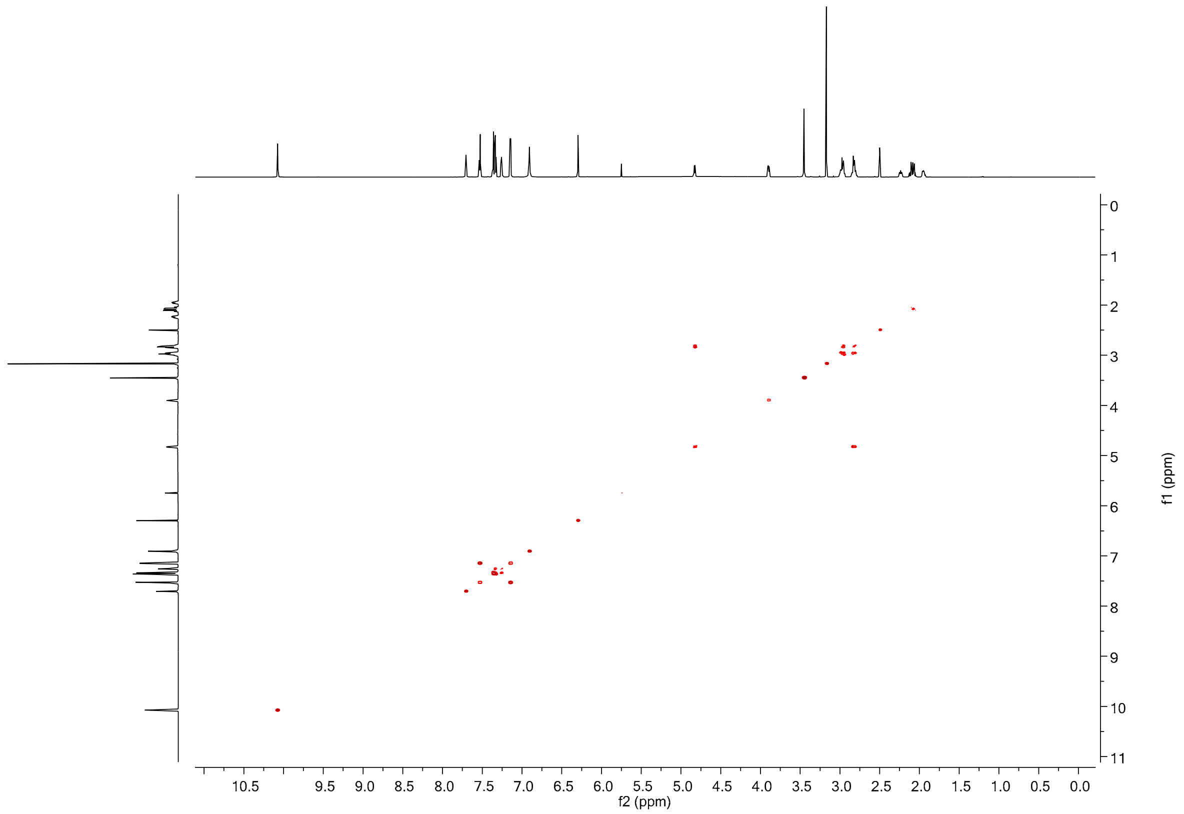

Figure S14. Solution-state 2D (^1H-^1H COSY) NMR spectrum of MBR-PG co-amorphous (DMSO-d_6).

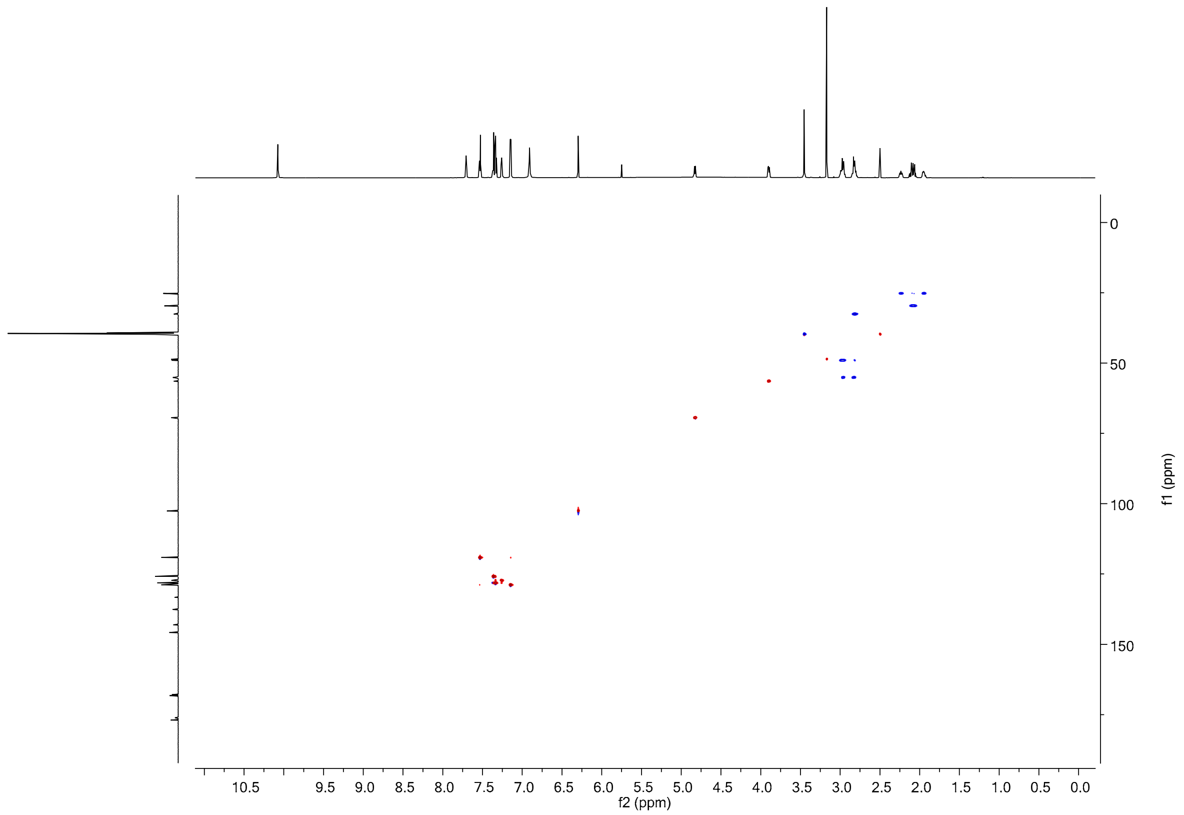

Figure S15. Solution-state 2D (^{1}H-^{13}C HSQC) NMR spectrum of MBR-PG co-amorphous (DMSO-d_6)

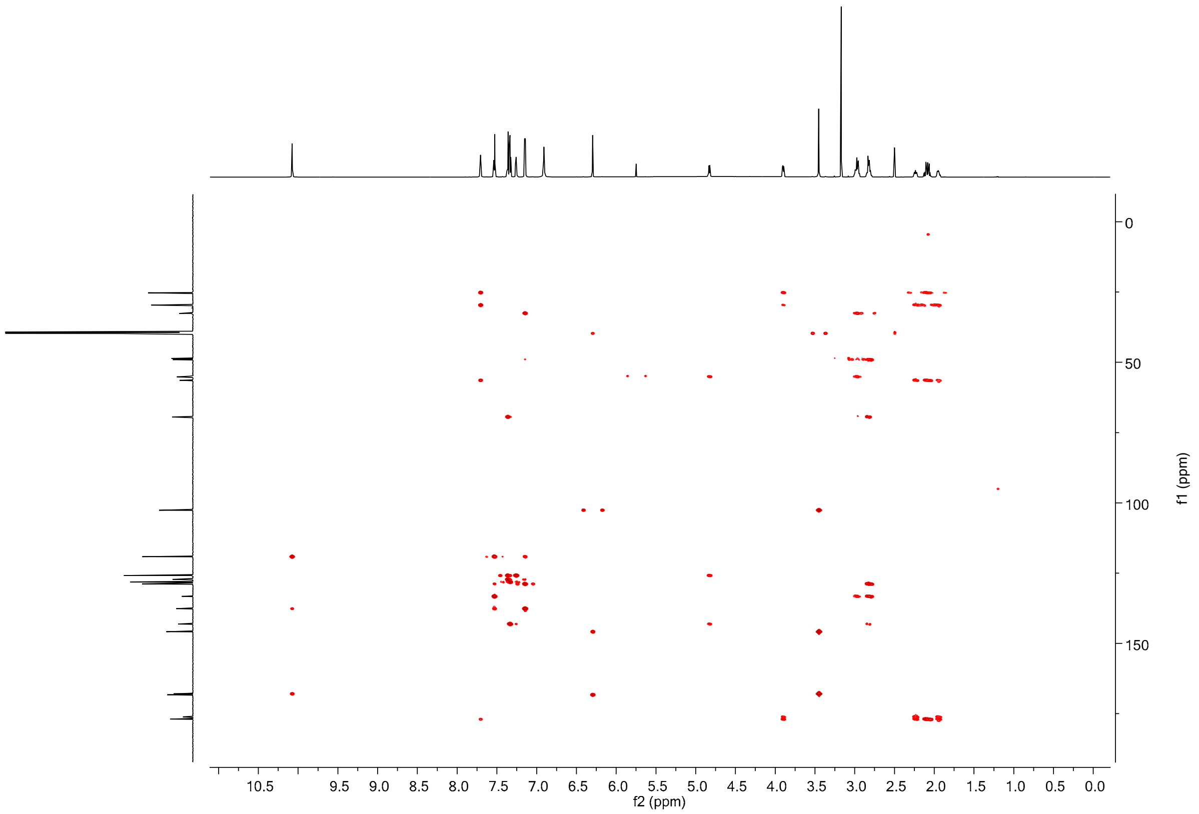

Figure S16. Solution-state 2D (^{1}H-^{13}C HMBC) NMR spectrum of MBR-PG co-amorphous (DMSO-d_6).

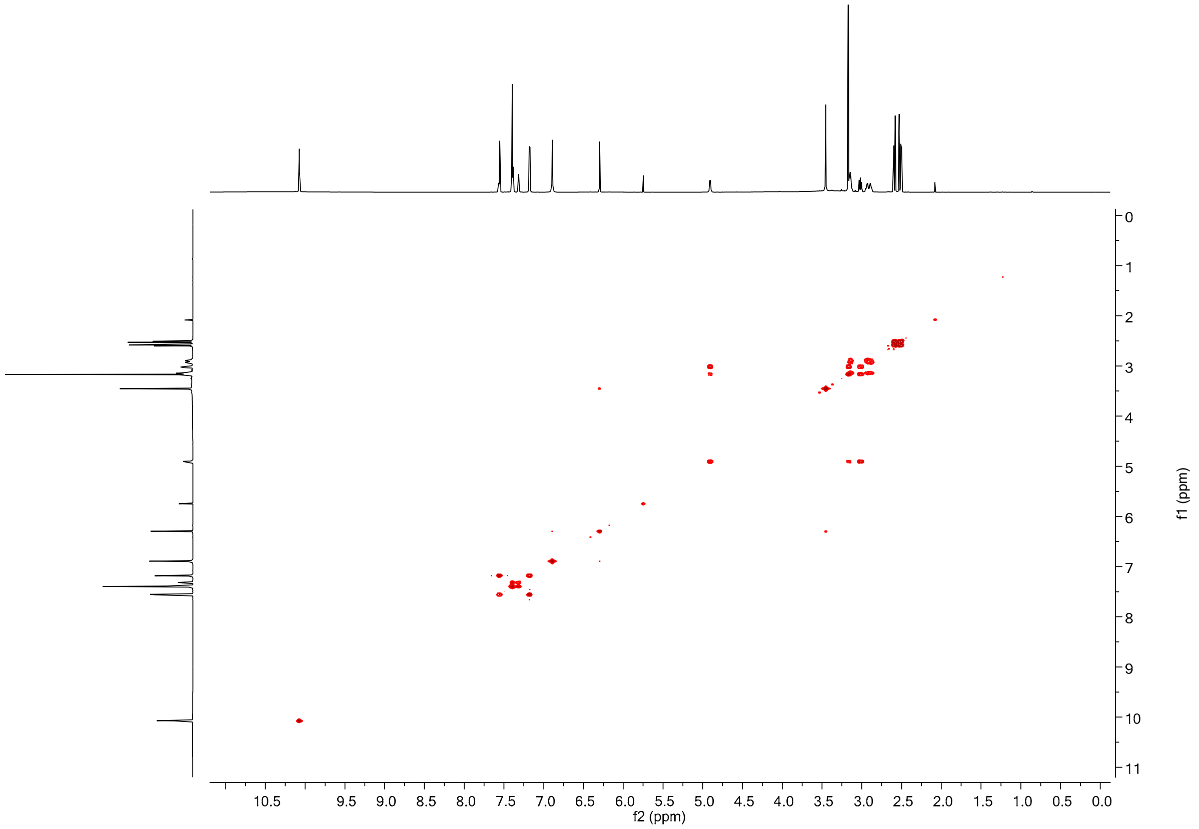

Figure S17. Solution-state 2D (^{1}H-^{1}H COSY) NMR spectrum of MBR-CA co-amorphous (DMSO-d_6).

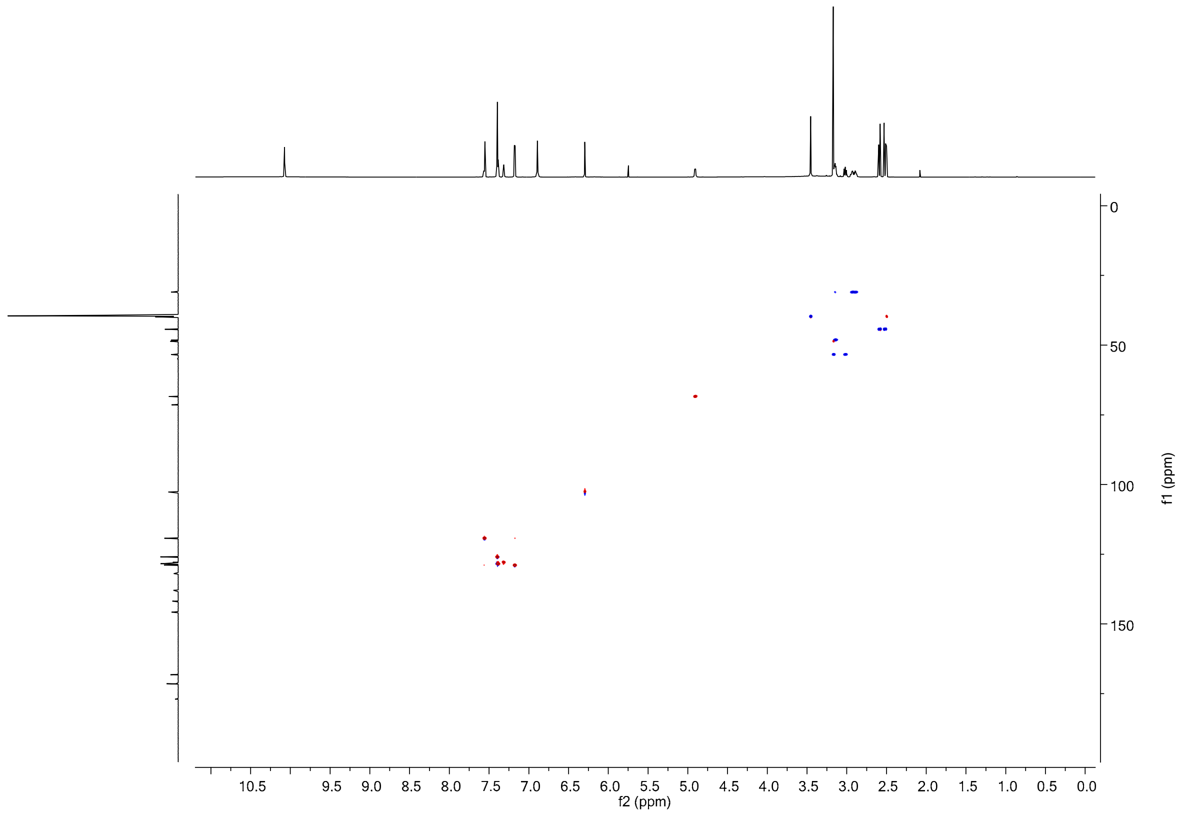

Figure S18. Solution-state 2D (^{1}H-^{13}C HSQC) NMR spectrum of MBR-CA co-amorphous (DMSO-d_6).

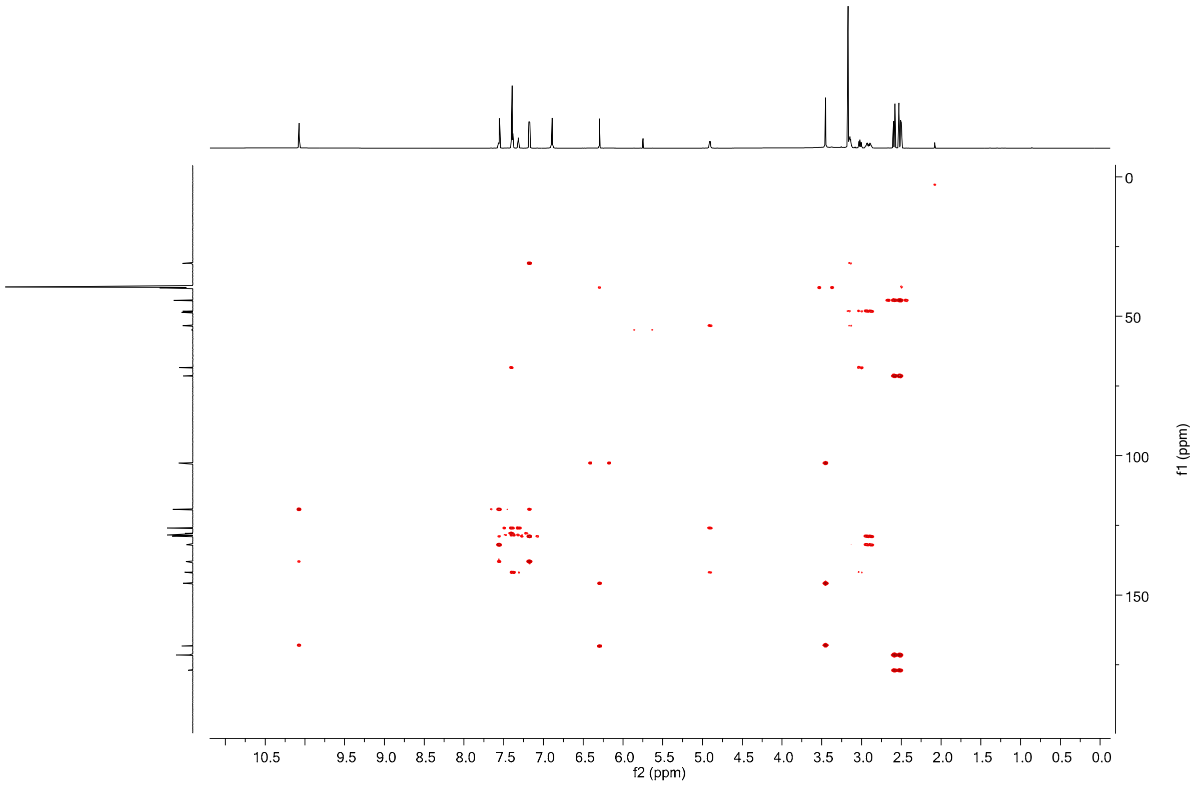

Figure S19. Solution-state 2D (^1H-^{13}C HMBC) NMR spectrum of MBR-CA co-amorphous (DMSO-d_6).

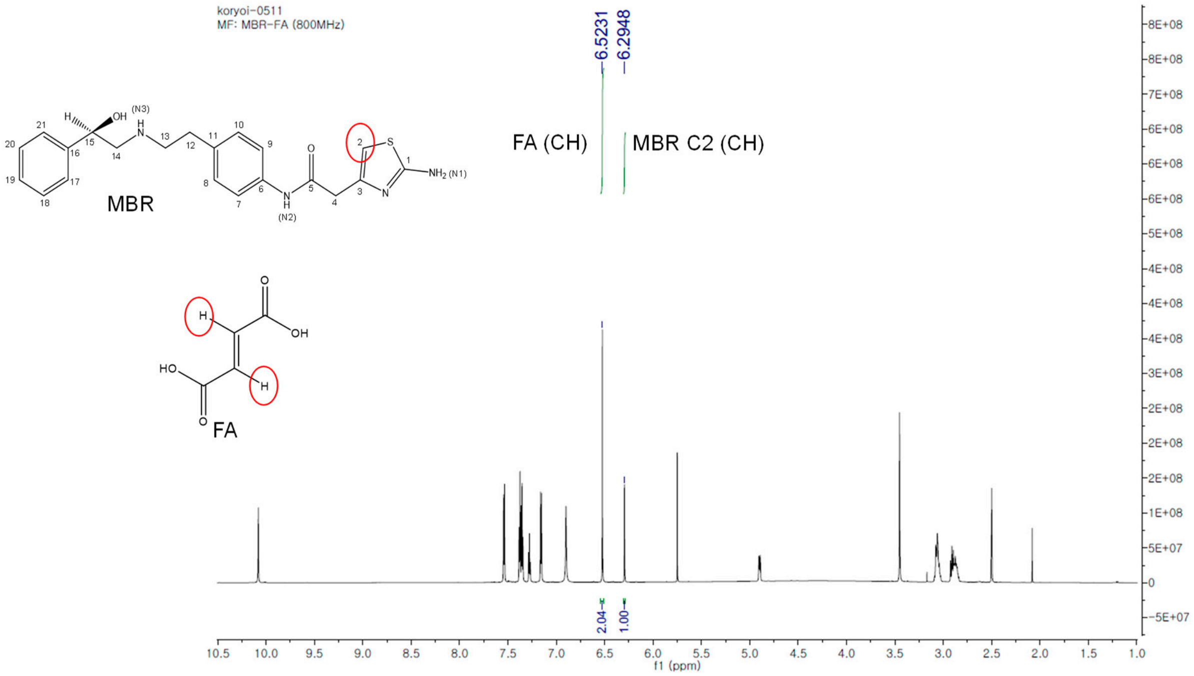

Figure S20. Solution-state ^1H-NMR spectrum of MBR-FA co-amorphous.

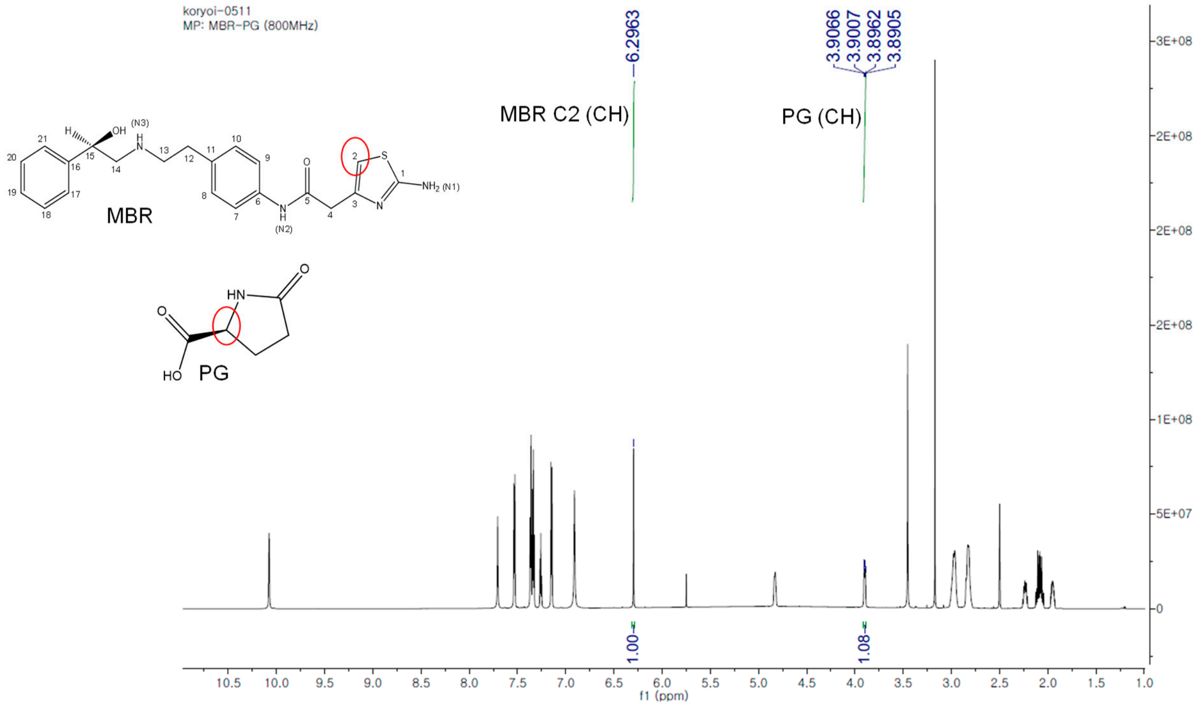

Figure S21. Solution-state ¹H-NMR spectrum of MBR-PG co-amorphous.

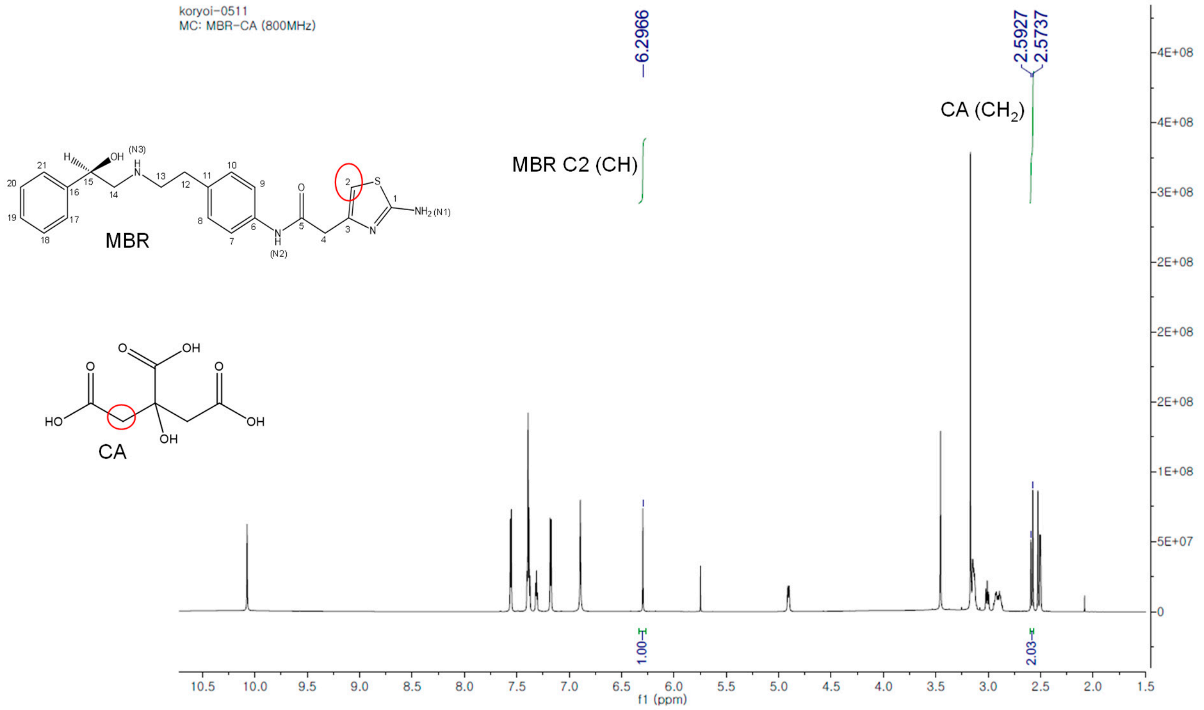

Figure S22. Solution-state ¹H-NMR spectrum of MBR-CA co-amorphous.

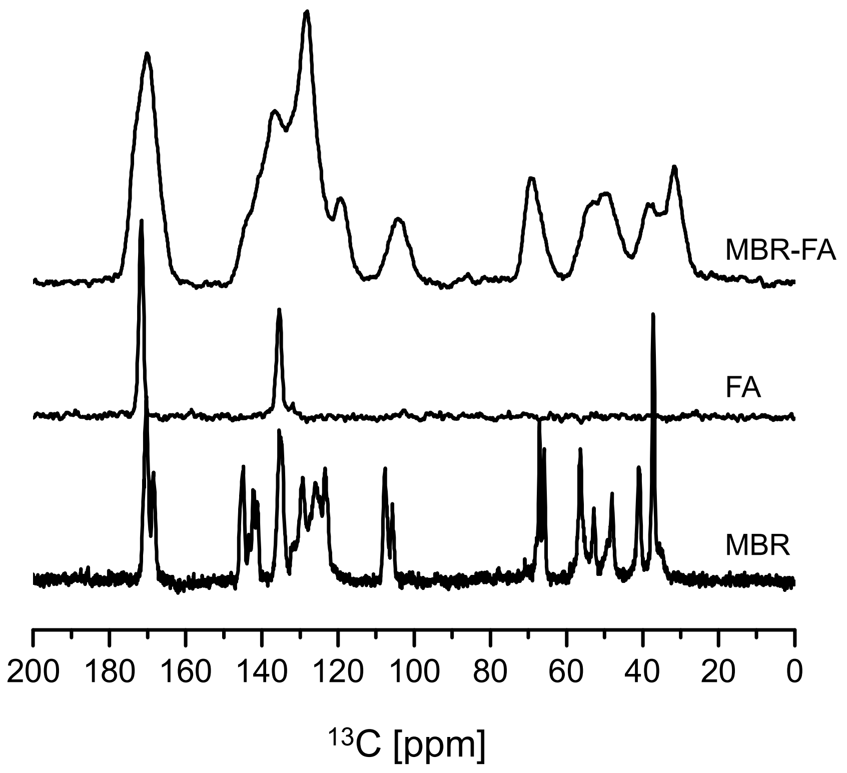

Figure S23. Solid-state CP/MAS ^{13}C-NMR spectra for MBR-FA co-amorphous, MBR and FA.

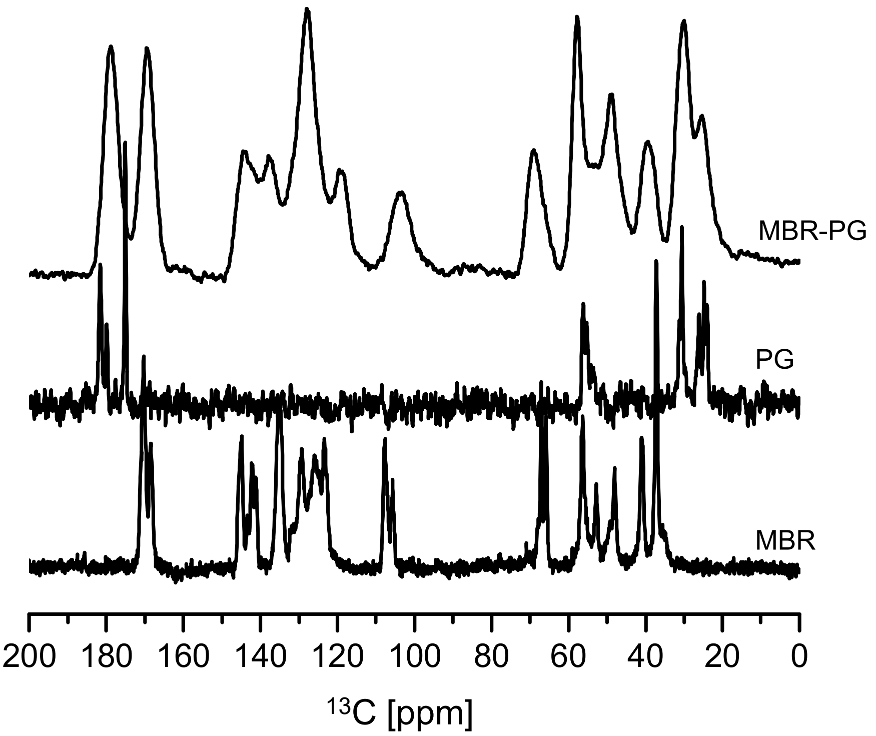

Figure S24. Solid-state CP/MAS ^{13}C-NMR spectra for MBR-PG co-amorphous, MBR and PG.

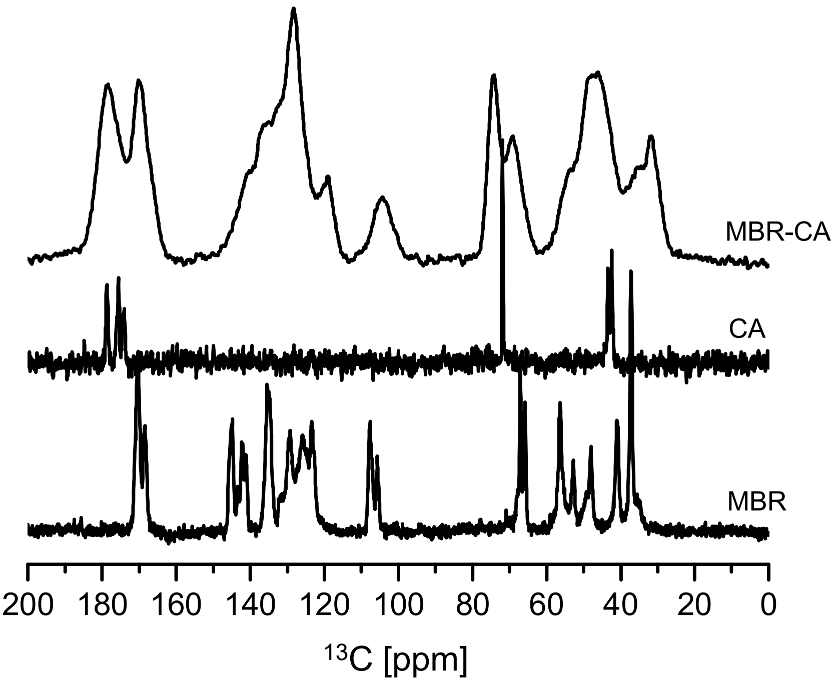

Figure S25. Solid-state CP/MAS ^{13}C-NMR spectra for MBR-CA co-amorphous, MBR and CA.

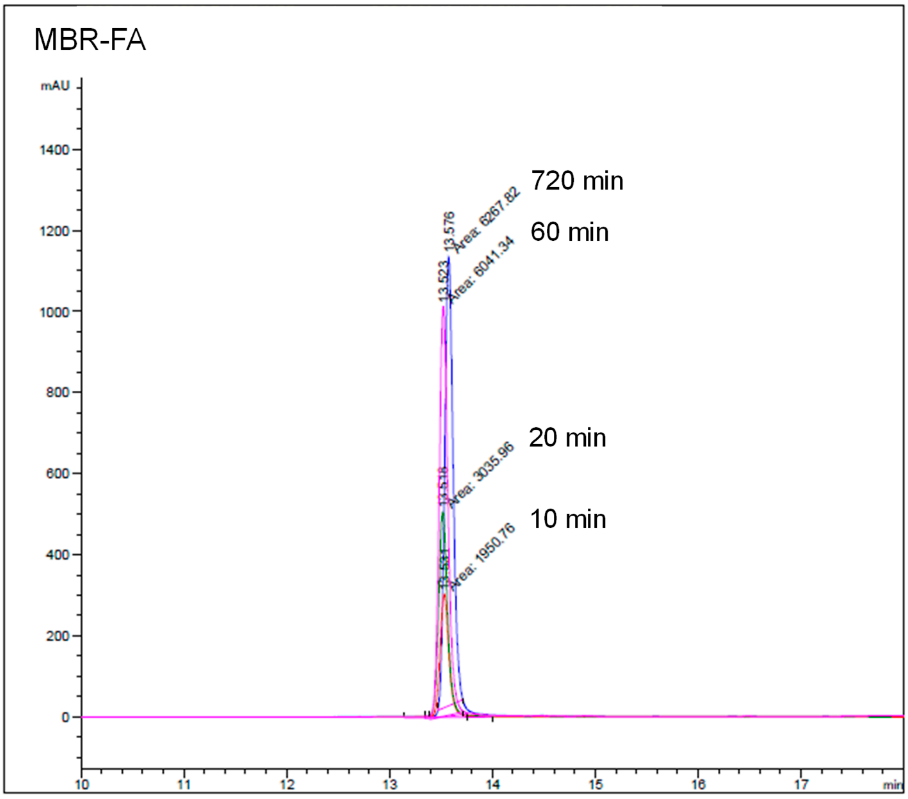

Figure S26. High-performance liquid chromatography (HPLC) chromatograms of the dissolution of MBR-FA at 10 min, 20 min, 60 min and 720 min.

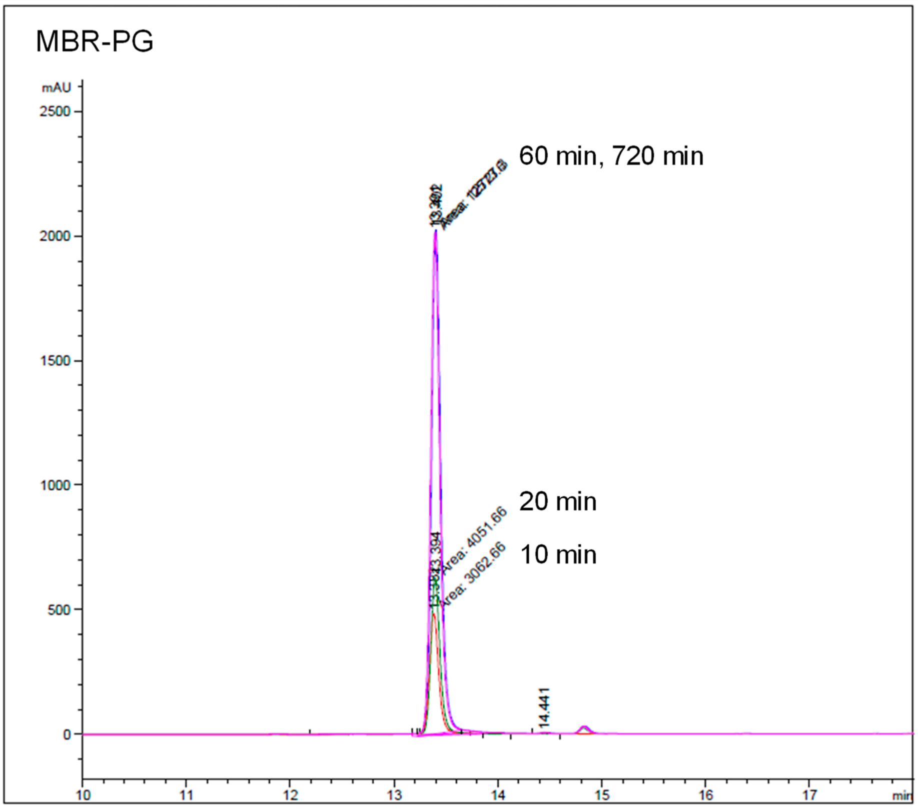

Figure S27. HPLC chromatograms of the dissolution of MBR-PG at 10 min, 20 min, 60 min and 720 min.

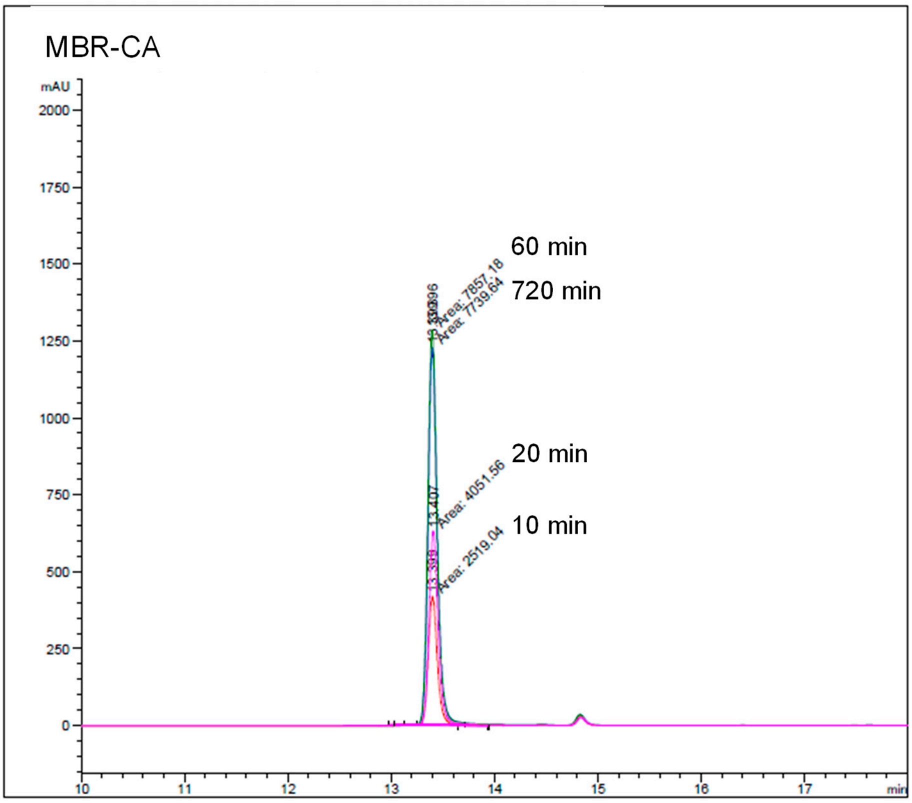

Figure S28. HPLC chromatograms of the dissolution of MBR-CA at 10 min, 20 min, 60 min and 720 min.
